# The latent organization of white matter microstructure and its relation to fluid intelligence

**DOI:** 10.1162/IMAG.a.1167

**Published:** 2026-03-19

**Authors:** Henrike M. Jungeblut, Erhan Genç, Michael Burke, Patrick D. Gajewski, Stephan Getzmann, Edmund Wascher, Anna-Lena Schubert

**Affiliations:** Department of Psychology, Johannes Gutenberg University, Mainz, Germany; Leibniz Research Centre for Working Environment and Human Factors, Dortmund, Germany; German Center for Mental Health (DZPG) partner site Bochum/Marburg, Bochum, Germany

**Keywords:** white matter microstructure, intelligence, structural equation modeling, fractional anisotropy, intra-neurite volume fraction, magnetization transfer ratio

## Abstract

White matter microstructure is a candidate neurobiological substrate underlying individual differences in fluid intelligence, potentially through differences in neural information transfer. Investigating the association between white matter microstructure and fluid intelligence requires precise modeling of these microstructural properties across the brain. Yet, it remains unclear whether MRI-derived markers of white matter microstructure generalize across tracts to support latent modeling approaches. Therefore, our primary objective was to derive measurement models for markers of white matter integrity (fractional anisotropy, FA), neurite density (intra-neurite volume fraction, INVF), and myelin content (magnetization transfer ratio, MTR) across 52 tracts (HCP-1065 atlas) grouped into 10 functional clusters. We investigated data of *N* = 365 individuals (age range: 18−74 years) drawn from two independent samples (Dortmund Vital Study: *N* = 150, Clinicaltrials.gov: NCT05155397; Mainz Network Study: *N* = 215). Confirmatory factor analyses consistently favored hierarchical bifactor models, capturing both a general factor per marker and orthogonal hemisphere-specific factors, independent of participants’ age. The general factors FA and MTR of the favored measurement models were significantly associated with fluid intelligence, assessed with matrix reasoning tests, FA: β = 0.26, *p* < .001 and MTR: β = 0.25, p = .017. When controlling for age, the association of fluid intelligence with FA remained significant, β = 0.14, *p* < .043, while the association with MTR was no longer significant, β = 0.11, *p* = .328. These findings establish anatomically informed measurement models for white matter microstructure and provide a scalable framework for investigating the biological underpinnings of cognitive abilities.

## 1 Introduction

Fluid intelligence is defined as the ability to use abstract reasoning in order to solve novel problems that do not depend on task-specific, cultural, or educative knowledge but rather on biological factors (e.g., Carroll, 1993; Horn & Cattell, 1966). Despite being one of the most defining traits of human cognitive abilities, its exact biological underpinnings remain a subject of ongoing debate. Microstructural properties of the white matter such as its integrity, the degree of myelination, and the nerve fiber density and orientation affect the quality and speed of information transmission along axons and likely play a crucial role in shaping interindividual differences in fluid intelligence. According to the neurocognitive process model (Schubert & Frischkorn, 2020), microstructural variation shapes intelligence indirectly by influencing mental speed, as reflected in neural (e.g., latencies of event-related potentials, ERPs) and behavioral (e.g., reaction times) measures. Similarly, the watershed model of individual differences in fluid intelligence (Kievit et al., 2016) views fluid intelligence as the downstream end product of multiple small causal influences such as variation in white matter microstructure that acts via intermediate variables such as mental speed. Prior research has consistently shown that fractional anisotropy (FA), a measure for the structural integrity of the white matter, is related to mental speed and intelligence (Booth et al., 2013; Ferrer et al., 2013; Fuhrmann et al., 2020; Genç & Fraenz, 2021; Kievit et al., 2016; Penke et al., 2012; Stammen et al., 2023; Tamnes et al., 2010; Wendelken et al., 2017). Here, we derive the latent structure of FA alongside two additional markers, intra-neurite volume fraction (INVF) and magnetization transfer ratio (MTR), to test the core prediction of these models hypothesizing that higher expression of these white matter microstructural markers is associated with higher fluid intelligence.

FA is a popular measure of white matter integrity that is commonly used in the field of intelligence research. However, FA does not directly measure white matter integrity but the main orientation of diffusion in each voxel of a diffusion-weighted MR image, making it an unspecific marker. The FA marker cannot distinguish between potential contributors to directional diffusion, such as neurite density or myelin content as also recently acknowledged in the field of intelligence research (Stammen et al., 2025). To address this limitation, we included a marker for neurite density (intra-neurite volume fraction, INVF) obtained by the diffusion MRI technique neurite orientation dispersion and density imaging (NODDI, Zhang et al., 2012) as well as a marker for myelin content (magnetization transfer ratio (MTR), Wolff & Balaban, 1989) obtained by quantitative MRI (qMRI). INVF is one of several markers provided by the NODDI model, which distinguishes intra-cellular, extra-cellular, and cerebrospinal fluid compartments (Zhang et al., 2012). The INVF marker can explain observed increases (or decreases) in FA as an increased (or decreased) neurite density within the white matter (Beaulieu, 2010) and has been shown to be positively associated with a general factor of intelligence in several white matter tracts (Stammen et al., 2025). MTR describes the interaction between mobile protons in water and motionally restricted protons such as in myelin (Vavasour et al., 2011) and is often used as an estimate for myelin content as it is reduced in progressing demyelinating diseases such as multiple sclerosis (Gass et al., 1994) and animal models of white matter diseases (Dousset et al., 1992). Additionally, low post-mortem myelin content is associated with decreased MTR (Schmierer et al., 2004). Following this, MTR can explain observed increases (or decreases) in FA as an increased (or decreased) myelin content. Hereafter, we use the terms white matter integrity and FA interchangeably. We use the terms neurite density and myelin content to refer to the more specific markers INVF and MTR, respectively.

To leverage these complementary markers in cognitive neuroscience, it is essential to understand how microstructural properties are organized across tracts and whether they support latent factors that relate to intelligence. However, research on the factor structure of white matter microstructure across tracts remains scarce and inconclusive, and to date, it remains unknown whether MRI-derived markers of white matter microstructure generalize across a variety of tracts, allowing for the modeling of general factors that reflect brain-wide properties. Some findings suggest that general factors for MRI-derived markers of white matter microstructure can be extracted from a series of white matter tracts (Cox et al., 2016; Penke et al., 2012). In contrast, other findings indicate that markers of white matter microstructure cannot be reduced to a single dimension (Fuhrmann et al., 2020; Kievit et al., 2016; Laukka et al., 2013; Lövdén et al., 2013). Alternative models have been proposed, including those that treat tract-average estimates as independent manifest variables that are correlated with fluid intelligence (Fuhrmann et al., 2020; Kievit et al., 2016), and those that capture shared variance between bilateral tracts using correlated tract-specific latent variables (Laukka et al., 2013; Lövdén et al., 2013). The heterogeneity in the description of the white matter factor structure may stem from differences in study populations, especially the mean age and the age ranges of participants, because white matter microstructure becomes increasingly correlated across tracts with aging (Cox et al., 2016), potentially favoring single-factor solutions in older and multi-factor solutions in younger populations. Additionally, methodological factors such as the choice of the MRI-derived markers (commonly FA, with the markers INVF, MTR, and T1 longitudinal relaxation used less frequently), the tract atlas, the tracts and number of tracts included (number of tracts in the reviewed literature ranges between 12 and 27), and whether tracts are averaged bilaterally potentially explains the diversion of results.

### 1.1 The present study

Understanding individual differences in cognitive abilities requires precise modeling of white matter microstructure across the brain. This study systematically addresses longstanding uncertainties about the dimensionality of MRI-derived microstructural markers, while explicitly accounting for key sources of heterogeneity that have limited previous research. To achieve this, we analyzed a combined data set of two independent adult samples (Dortmund Vital Study, Clinicaltrials.gov: NCT05155397; Mainz Network Study), examining three MRI-derived markers, namely FA, INVF, and MTR. Due to differences in acquisition protocols, MTR data were available only for the Dortmund sample. Crucially, by including less frequently studied markers alongside FA, we aimed to overcome the biological non-specificity of conventional diffusion metrics.

To maximize neuroanatomical coverage, we employed a comprehensive tract atlas of 52 association and projection fibers. Using a preregistered, theory-guided confirmatory factor analysis (CFA) framework, we systematically compared competing measurement models: single-factor models (Cox et al., 2016; Penke et al., 2012), manifest-variable models (Fuhrmann et al., 2020; Kievit et al., 2016), tract-specific latent variable models (Laukka et al., 2013), and novel bifactor models incorporating hemispheric structure.

This systematic modeling strategy allowed us to identify general and hemisphere-specific factors for each marker, improving upon earlier approaches that largely treated white matter microstructure as unidimensional. Structural equation modeling (SEM) further enabled the integration of multiple markers while accounting for measurement error. Finally, we incorporated fluid intelligence as an outcome to test the central hypothesis that higher global white matter integrity supports higher fluid intelligence.

### 1.2 Hypotheses

Rather than proposing a specific hypothesis about the dimensionality of white matter microstructure, we tested multiple competing models to determine which best captured the covariance structure of the data. We hypothesized that individual differences in white matter microstructure would significantly relate to differences in fluid intelligence within a latent variable framework. Specifically, we expected that individuals with higher fluid intelligence would exhibit greater white matter integrity, neurite density, and myelin content, reflected in higher FA, INVF, and MTR values, respectively.

## 2 Materials and Methods

We analyzed a combined data set made of two independent data sets. The analysis procedure was preregistered (https://osf.io/8kuxz) and initially planned for the Dortmund data only. However, data from the Mainz Network Study became available earlier than expected, allowing us to incorporate this additional sample to increase our sample size, thereby enhancing our statistical power and mitigating concerns related to the moderate sample size of the Dortmund sample. Below, we summarize the key materials and analysis steps, along with any deviations from our preregistration. Readers interested in more detailed descriptions are encouraged to refer to the full preregistration.

### 2.1 Sample

The combined data set includes data of *N* = 365 individuals (164 males, 201 females) with an average age of *M* = 35.66 years (*SD* = 14.48, range: 18−74). Below, we summarize the key demographic and acquisition details for the two independent samples.

#### 2.1.1 Dortmund Vital Study

Data of *N* = 150 participants (62 males, 88 females) with a mean age of *M* = 47.05 years (*SD* = 14.99, range: 20–74) were drawn from the ongoing cross-sectional and longitudinal Dortmund Vital Study (Gajewski et al., 2022). In this sample, 81.33% of participants were employed at the time of data acquisition, while the majority of participants who were not employed were retired. In total, 50% of participants held a university degree including PhD degrees, 27.33% held a high school diploma, 18% had a lower secondary school-leaving certificate, and 3.33% had a primary school-leaving certificate.

Participants were recruited via an internet site, newspaper advertisements, reports and announcements in local print and radio media, public information events, social media, and flyers. Local companies were contacted and asked to distribute information on study participation. For the present analysis, we used intelligence test and MRI data from the second acquisition wave that began in late 2021. Subjects received 130 € for their participation in the second acquisition wave. Since this wave is still ongoing, we included all data that was collected up to the point of the first statistical analysis. Informed consent was obtained from all participants for being included in the study. The Dortmund Vital Study was conducted with approval from the local ethics committee of the Leibniz Research Centre for Working Environment and Human Factors at the Technical University of Dortmund.

#### 2.1.2 Mainz Network Study

We acquired data of *N* = 215 participants (102 males, 113 females) with a mean age of *M* = 27.71 years (*SD* = 6.76, range: 18−40) between March 2024 and February 2025. In this sample, 51.63% of people were employed and 33.95% of people were students at a university. In total, 5.12% of participants in this sample were holders of a PhD degree, 44.19% held a university or university of applied sciences degree, 45.12% had acquired the A-Levels or a vocational diploma, and 5.58% had a lower secondary school-leaving certificate or no school-leaving certificate.

Participants were recruited via an internet site, newspaper advertisements, reports in local radio media, social media, and flyers. Subjects received 100 € for their participation in all three of the study’s measurement sessions. For this analysis, we used intelligence test data (available for the whole sample) and MRI data (available for *n* = 200) collected at two different measurement sessions. All study participants gave informed consent to be included in the study. The study was approved by the local ethics committee of the Department of Psychology of the Johannes Gutenberg University Mainz.

#### 2.1.3 Sample size rationale

Data for this study were drawn from two independent samples and collected as part of two larger research projects. Consequently, we conducted post hoc power analyses using the R package *semPower* (Moshagen & Bader, 2023). This was done separately for the marker-specific measurement models for the FA and INVF markers estimated in the combined sample (*N* = 365) and the measurement models for the MTR marker estimated in the Dortmund sample (*N* = 150) (see below). We calculated the achieved power to detect a predefined degree of misfit (root mean square error of approximation (*RMSEA*) of ≥.05) for the measurement model with the fewest degrees of freedom (*df* = 27 for the FA and INVF models and *df* = 26 for the MTR models) at α = .05. The FA and INVF models reached a power of 1 − β = 0.82, indicating sufficient sensitivity to evaluate their fit. In contrast, the MTR models showed limited power (1 − β = 0.34). The reader should note that the power calculations are based on the RMSEA only. In our systematic model comparison, we rely on Akaike weights that compare competing models against each other for model selection, thereby enabling model selection even when the power of absolute goodness-of-fit indices is limited.

To estimate the required sample size for the latent correlation between white matter microstructure and fluid intelligence, we considered both the internal consistencies of the latent constructs and the expected population correlations between them, as reported in previous work (Kretzschmar & Gignac, 2019). Measures of FA, INVF, and qMRI-derived indices, as well as performance on Raven’s Progressive Matrices 2 and Raven’s Advanced Progressive Matrices, showed good to excellent reliability in previous studies. This has been established using intra-class correlations (ICC, Andica et al., 2020; Boekel et al., 2017; Edde et al., 2023; Schwartz et al., 2019) and item response theory (IRT)-based marginal reliabilities (Raven & Raven, 2003) or Chronbach’s α and test–retest reliabilities (Heller et al., 1998), respectively. Previous estimates of the latent correlation between a general factor FA and general or fluid intelligence range between *r* = .13 and *r* = .26 (Booth et al., 2013; Penke et al., 2012; Tamnes et al., 2010), while estimates of the latent correlation between a general factor INVF or MTR and general or fluid intelligence rarely exist. Assuming internal consistencies of ω = .90 (or ω = .80) in the current study and a population correlation of *r* = .20, 280 (or 360) observations are needed to reliably determine the latent relationship at a level of confidence of .80. Thus, the combined data with *N* = 365 participants meet the requirement to reliably determine the latent relationships between white matter microstructure and fluid intelligence.

### 2.2 Image acquisition and preprocessing

Both MRI data sets were acquired on 3 Tesla Magnetom Prisma scanners (Siemens Medical Solutions, Erlangen, Germany) using 64-channel head coils for signal reception. In the Dortmund Vital Study, diffusion-weighted images were acquired with the following parameters: repetition time (TR) = 2426 ms, echo time (TE) = 87 ms, flip angle = 90°, 64 slices, field of view (FOV) = 220 mm, voxel size = 2 × 2 × 2 mm. Diffusion weighting was based on a multi-shell, high-angular-resolution diffusion imaging (HARDI) scheme with b-values of 0, 1000, 1800, and 2500 s/mm^2^, applied along 20, 40, and 60 uniformly distributed directions (Genç et al., 2018). The set of orthogonal diffusion gradient directions was generated using the MASSIVE toolbox (Froeling et al., 2017). Eight volumes with no diffusion weighting (b = 0 s/mm^2^) were acquired for motion correction and computation of NODDI coefficients. In the Mainz Network Study, the diffusion-weighted sequence protocol was as follows: TR = 2620 ms, TE = 98.80 ms, flip angle = 90°, 64 slices, FOV = 220 mm, voxel size = 2 × 2 × 2 mm. Diffusion weighting was based on a multi-shell, soma, and neurite density imaging (SANDI) scheme with b-values of 0, 200, 500, 1000, 1800, 2500, 4000, and 6000 s/mm^2^, applied along 235 directions. The set of diffusion gradient directions was generated by MRtrix’ *gen_scheme* (www.mrtrix.org). Additionally, six volumes without diffusion weighting were acquired.

Magnetization transfer-(MT-) weighted images were acquired as part of the Dortmund Vital Study only and are not available in the Mainz data. The qMRI measurement protocol was based on Weiskopf et al. (2013). Three multi-echo fast low angle shot (FLASH) scans were acquired with predominant T1-, proton density- (PD-), and MT-weighting. The MT-weighted images were acquired with TR = 23.7 ms and flip angle = 6° and an off-resonance Gaussian-shaped radio frequency (RF) pulse (4 ms duration, 220° nominal flip angle, 2 kHz frequency offset from water resonance) was applied. Multiple gradient echoes were acquired with alternating readout polarity at four or six equidistant TEs (between 2.2 and 14.7 ms, most participants’ data were acquired with four echoes while some were acquired with six echoes after a protocol change). Other parameters were as follows: 1 mm isotropic resolution, 176 sagittal partitions, FOV = 256 × 240 mm, matrix = 256 × 240 × 176, parallel imaging using generalized autocalibrating partially parallel acquisitions (GRAPPA) factor 2 in phase-encoding (PE) direction, 6/8 partial Fourier in partition direction, non-selective RF excitation, readout bandwidth BW = 425 Hz/pixel, RF spoiling phase increment = 50°.

The preprocessing of the DWI data was conducted using FSL version 6.0.7.6. The preprocessing included the correction of susceptibility-induced distortions using FSL’s *topup* and a method similar to that described in Andersson et al. (2003). We corrected for eddy currents using FSL’s *eddy* (Andersson & Sotiropoulos, 2016). We visually checked the preprocessed data for excessive motion and scanner or field-related artifacts and excluded subjects if data quality was unsatisfactory (MRI data of *n*= 2 participants from the Dortmund data were excluded due to strong artifacts and failure of non-linear registration, respectively). FA maps were calculated using FSL’s *DTIFIT*. For the extraction of NODDI coefficient maps, we used the accelerated microstructure imaging via convex optimization toolbox (AMICO, Daducci et al., 2015). The preprocessing of the qMRI data and the calculation of MTR maps was done using the hMRI toolbox (Tabelow et al., 2019) in SPM12 (https://www.fil.ion.ucl.ac.uk/spm/software/spm12/) in MATLAB version R2023b (The MathWorks, Inc.). We performed B1+ bias correction using the *rf_map* method and RF sensitivity bias correction using body and head coil measurements.

### 2.3 Microstructure of the white matter

To derive the tract-average FA, INVF, and MTR values, we binarized and thresholded 52 white matter tracts defined by the HCP-1065 probabilistic tract atlas (Yeh, 2022) at a threshold of 0.8 to reduce tract overlapping. We used nonlinear registration with the nearest neighbor interpolation to bring each tract into the individual participant’s T1w space. We then used FSL’s *epi_reg* to generate a white matter segmentation and obtain the transform matrix to linearly register the images to the individual participants’ diffusion or MTR space. We first masked the tracts to include white matter voxels only using the previously generated white matter segmentation and then applied the linear transform using nearest neighbor interpolation. We visually inspected the transformed tracts to ensure good registration results by overlaying them onto the individual subjects’ FA, INVF, or MTR images and checking for good white matter coverage. We masked the marker maps by each white matter tract and averaged across tracts, yielding an average FA, INVF, and MTR value per subject per tract.

We discarded any FA, INVF, or MTR values exceeding ±3 standard deviations from the Dortmund sample’s mean or the Mainz sample’s mean, respectively. In the Mainz data, 1.06% of all INVF values were removed. For all other variables in both data sets, less than 1% of all values were removed. We then *z*-standardized the data within the two samples before merging the two data sets and *z*-standardizing again across the combined data.

### 2.4 Behavioral outcome variable: Fluid intelligence

In the Dortmund Vital Study, fluid intelligence was measured using the pen-and-paper form of the Raven’s Progressive Matrices 2 (Raven & Raven, 2003) and operationalized as the number of correctly solved items by the total number of items. The Raven’s Progressive Matrices 2 is a nonverbal test requiring little verbal instruction. The test is a revised and integrated version of earlier Raven’s Progressive Matrices series, and incorporates the Raven’s Coloured Progressive Matrices (Raven, 1947), the Raven’s Standard Progressive Matrices (Raven, 1938), and the Raven’s Advanced Progressive Matrices (Raven, 1962), thus allowing one to assess cognitive abilities in samples of broad ranges of intellectual capabilities and ages. The test was administered individually for each participant. Participants completed 48 items (item sets B, C, D, and E) within a 45-minute time limit, selecting the correct answer from five possible options. No subject scored at or below chance level, which was the preregistered exclusion criterion for fluid intelligence test data. On average, participants correctly answered 64.44% (*SD* = 14.65 %) of the items, corresponding to a mean IQ of *M* = 112.29 (*SD* = 16.10). It is important to note that the Raven’s Progressive Matrices 2 provides IQ norms up to the age of 69 years only. In this sample, *n* = 9 individuals were older than 69 years, meaning their IQ scores may be underestimated. The test showed excellent odd–even reliability computed as the Spearman–Brown-corrected correlation of accuracies of odd and even items, *r*__odd−even__ = 0.95.

In the Mainz Network Study, we used a digitized version of the German Advanced Progressive Matrices (Heller et al., 1998) to measure participants’ fluid intelligence. The Advanced Progressive Matrices are a more challenging version than the Raven’s Standard Progressive Matrices and are designed to better differentiate among higher-ability individuals. We selected this test because the study sample included many university students and graduates, resulting in an overrepresentation of individuals with high educational attainment. Again, we operationalized fluid intelligence as the number of correctly solved items by the total number of items. The Advanced Progressive Matrices were administered in groups of up to seven individuals as part of a battery comprising eight cognitive tests during the intelligence and working memory assessment session. Participants were presented with 36 items (item set 2) and instructed to select the correct answer from 8 possible options. The recommended time limit of 40 minutes was not imposed, participants had unlimited time for completion. This decision was motivated as the Mainz Network Study also included the Berlin Intelligence Structure Test (not reported here, Jäger et al., 1997), which is a speeded measure of general intelligence. The Advanced Progressive Matrices were included as an unspeeded counterpart to balance the assessment of intelligence and ensure that performance reflected reasoning ability rather than speed. No subject scored at or below chance level. Participants correctly answered 74.51% (*SD* = 16.34%) of the items. The samples’ mean IQ was *M* = 102.23 (*SD* = 18.28). It is important to note that the average IQ may be slightly overestimated because the normative data are based on the standard 40-minute time limit version of the test. In the present sample, the test showed good odd–even reliability, *r*__odd−even__ = 0.87.

To combine the fluid intelligence data from the two data sets, we *z*-standardized the data within each sample before merging the two data sets and *z*-standardizing again across the combined data.

### 2.5 Confirmatory factor analysis

In brief, we examined the factor structure of different white matter microstructure markers by testing several competing confirmatory factor models (see below) against one another. After selecting a winning measurement model for each of the microstructure markers, we investigated their latent relationship by combining them into a comprehensive white matter microstructure model. Specifically, we tested whether a multi-factor model with correlated marker-specific general factors, or a hierarchical model with a higher-order general factor extracted from the general factors FA, INVF, and MTR, best fit the data and again used model comparison to identify the winning model. It is possible that the FA, INVF, and MTR measures extracted from the same cluster of tracts share commonalities that bias the latent associations between marker-specific general factors. To account for these sources of covariance, we added orthogonal method factors per white matter tract cluster to the multi-factor and hierarchical solutions and assessed whether this modification improved model fit. Method factors help capture measurement-related influences that arise when variables share common sources of variability that are unrelated to the core constructs of interest. In this case, sampling different markers from the same white matter tract cluster may introduce shared effects that possibly inflate the true relationships between FA, INVF, and MTR. Finally, we included a dependent latent variable fluid intelligence into the winning white matter microstructure model to investigate the latent relationship between markers of white matter microstructure and fluid intelligence.

In all models, we accounted for the potential confound *data source* by including a dummy variable that coded whether a data point belonged to the Dortmund or the Mainz sample. This dummy variable was included as a regressor pointing to all exogenous latent variables and the residuals of all endogenous latent variables.

We used the R package lavaan (Rosseel, 2012) and the full-information maximum likelihood estimator to specify marker-specific measurement models in RStuido version 4.3.3. We examined all manifest variables for univariate normality by inspecting skewness and kurtosis. All values remained below the threshold values of skewness = 2 and kurtosis = 7 (West et al., 1995) that we specified in our preregistration. For model identification, we fixed the variances of all latent variables to 1 and freely estimated all factor loadings. Whenever a latent variable loaded on two indicators only, we constrained the factor loadings to be equal to ensure local identifiability. If the variances of any manifest or latent variables were estimated to be negative and not statistically significant, we fixed the corresponding variance to 0.

For goodness-of-fit evaluation, we preregistered to use the Comparative Fit Index (CFI, Bentler, 1990) as well as the RMSEA (Browne & Cudeck, 1992). We decided to also include the Tucker–Lewis Index (TLI, Tucker & Lewis, 1973) and the Standardized Root Mean Square Residual (SRMR, Bentler, 1990) for a more fine-grained model assessment, because the CFI and RMSEA sometimes yielded contradictory conclusions. A *CFI*/*TLI* of ≥.90 and an *RMSEA*/*SRMR* of ≤0.08 indicate an acceptable model fit, while a *CFI*/*TLI* of ≥.95 and an *RMSEA*/*SRMR* of ≤0.05 suggest a good fit (Hu & Bentler, 1999). We also report χ^2^ model test results but did not use them as a formal test statistic since they are strongly dependent on sample size. For the systematic model comparison, we computed the relative fit index Akaike’s Information Criterion (AIC, Akaike, 1998) for each model and transformed the raw AIC values into Akaike weights, which were calculated relative to all other marker-specific models (Wagenmakers & Farrell, 2004). Akaike weights represent the conditional probabilities of each model, allowing us to identify the best-fitting model for each marker. For the evaluation of the significance of individual model parameters, we adopted a significance threshold of α = .05.

#### 2.5.1 Preregistered measurement models

In our preregistration, we specified 28 different measurement models for each of the markers FA, INVF, and MTR. These models varied along several dimensions. First, some allowed for the extraction of a general factor for each marker across all 52 tracts (used as indicator variables), while others did not. Second, we explored alternative classifications of white matter tracts, including a basic division into association and projection fibers, as well as more fine-grained functional clusters proposed by Yeh (2022). Finally, we specified a series of models with tract-specific latent variables capturing the common variance of corresponding tracts across hemispheres. A summary of all preregistered models is provided in Supplementary Table S1.

However, the majority of our preregistered models failed to converge, primarily due to multicollinearity arising from spatial overlap among tracts (see Supplementary Tables S2–S7 for full details). Given these issues, we discontinued the model comparison using the preregistered models, as the models did not adequately capture the structure of the data. Instead, we developed a series of modified models aimed at addressing the unexpected multicollinearity of several indicator variables.

#### 2.5.2 Modified and exploratory measurement models

**Fig. 1.**
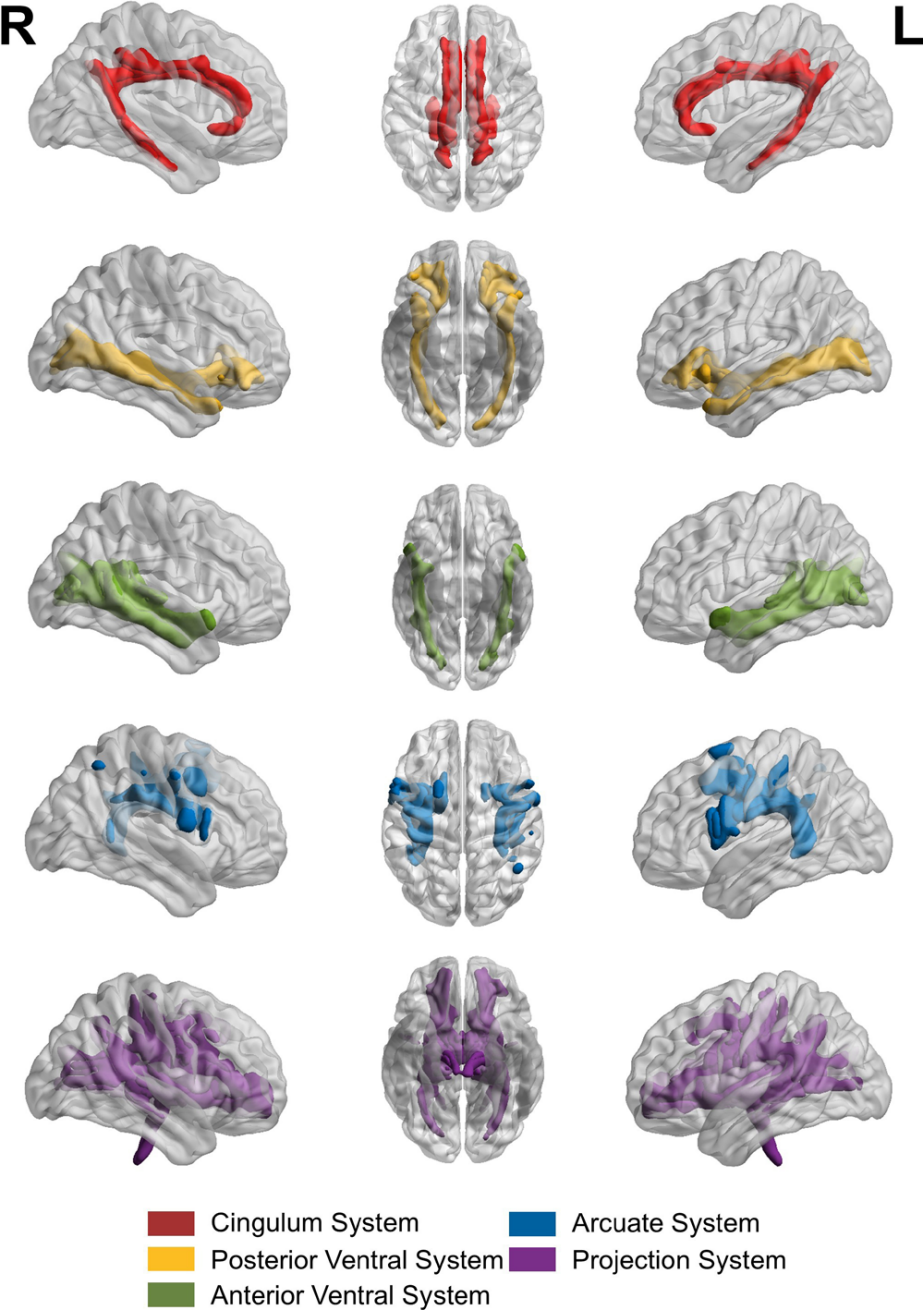
Functional tract clusters according to Yeh (2022). *Note*: The white matter tract clusters modeled for analysis. The tract clusters are composed from the individual binarized and thresholded tracts.

**Fig. 2.**
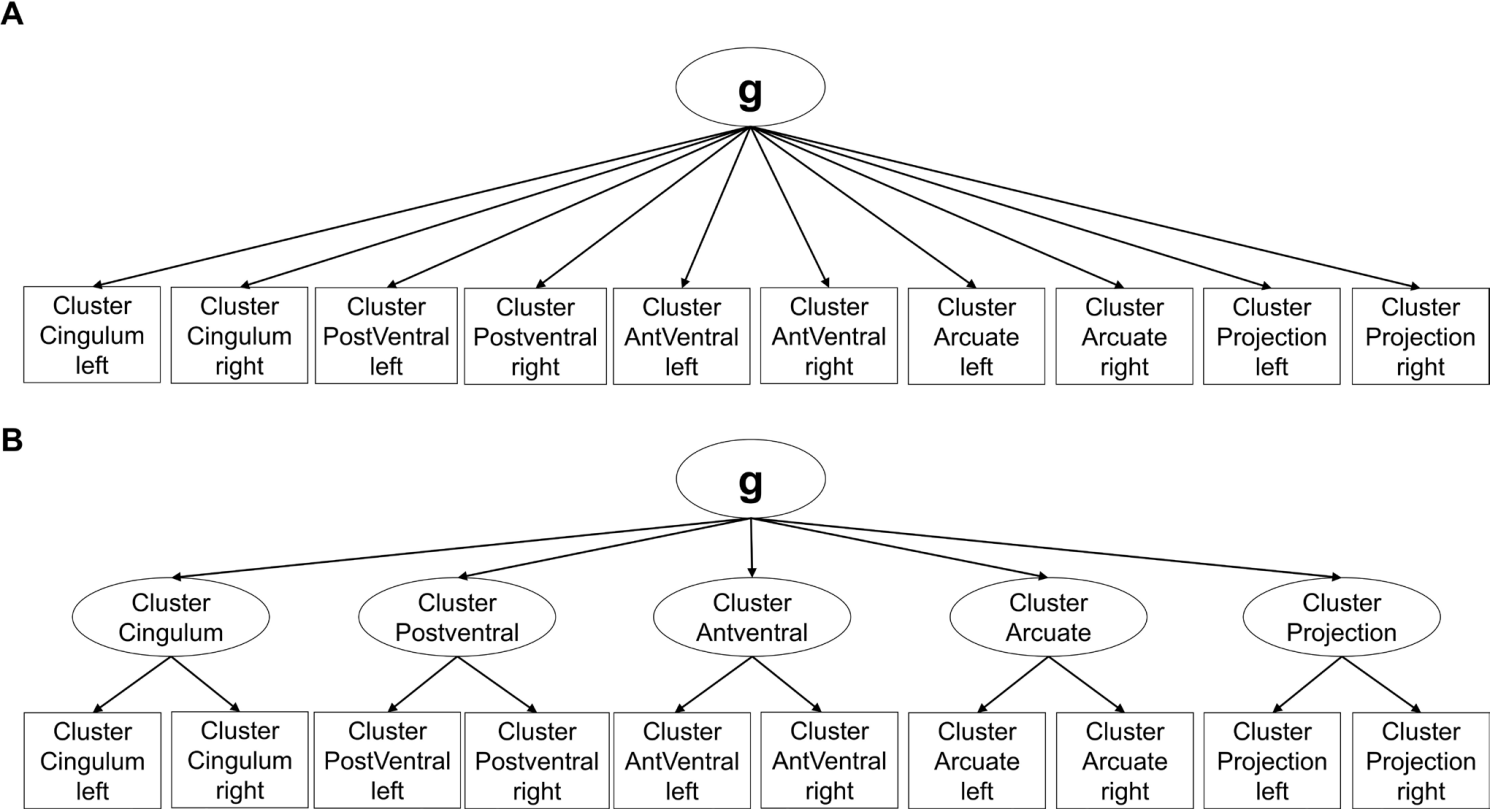
Exemplary measurement models. *Note*: In the Clustered Single-Factor Model (corresponds to the preregistered Single-Factor Model), a general factor loads onto the 10 functional cluster-average indicators (A). The Bilateral Clustered Single-Factor Model (corresponds to the preregistered Single-Factor Model with tract-specific latent variables) is a modification of the Clustered Single-Factor Model and includes bilateral latent cluster variables (B). Residual errors and the data source regressor are not depicted for simplification.

**Fig. 3.**
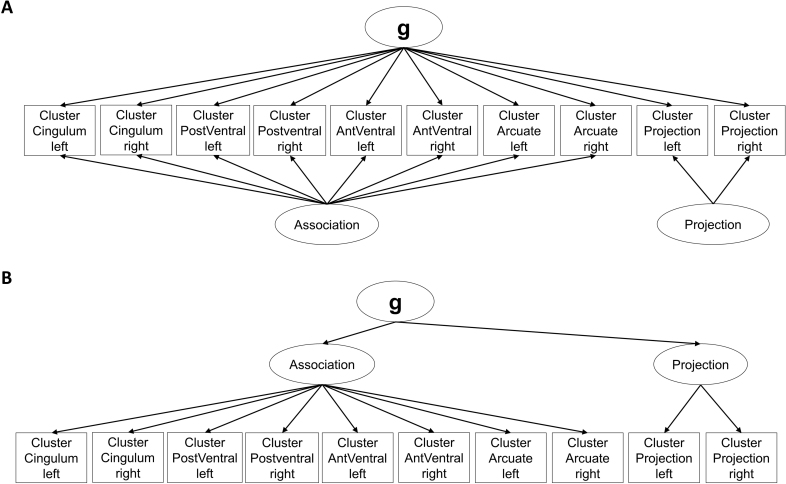
Exemplary measurement models with association and projection fiber division. *Note*: In the Clustered Bifactor Model (corresponds to the preregistered Bifactor Model), a general factor is included as a primary dimension and two latent variables (association and projection fibers) are included as a secondary dimension (A). In the Clustered Hierarchical Model (corresponds to the preregistered Hierarchical Model), the general factor loads onto a latent variable association fibers and a latent variable projection fibers, which in turn load onto the 10 functional cluster-average indicators (B). Residual errors and the data source regressor are not depicted for simplification.

The modified measurement models largely reflect the same underlying concepts as the preregistered models (see short description and visualizations of all models in Table 1 and Supplementary Figures S1–S6) and were run for the combined sample. The primary modification involved reducing the number of manifest indicators by averaging across tracts belonging to the same functional systems, as defined by Yeh (2022), to account for the spatial overlap of tracts. The included tract clusters (see Fig. 1) are the left and right cingulum system (frontal parahippocampal segment, frontal parietal segment, parahippocampal segment, parahippocampal parietal segment, and the parolfactory segment of the cingulum as well as the bilateral superior longitudinal fasciculus 1), the left and right posterior ventral system (uncinate fasciculus and inferior fronto-occipital fasciculus), the left and right anterior ventral system (middle longitudinal fasciculus, temporoparietal aslant tract, vertical occipital fasciculus, and inferior longitudinal fasciculus), and the left and right arcuate system (frontal aslant tract, superior longitudinal fasciculus 2 and 3, and arcuate fasciculus) as well as two clusters for the bundle of left and right projection fibers (the remaining 20 projection fibers from the atlas). Beyond this adjustment, we adhered to our preregistered approach of specifying models in which the indicator variables either allow (or do not allow) for the extraction of a general factor (see Fig. 2A). We also retained the models with a division of association versus projection fibers (see Fig. 3A, B). In line with the preregistered models that included tract-specific latent variables, we extended this approach by introducing cluster-specific latent variables that captured shared variance across hemispheres for the same functional cluster (see Fig. 2B for the Bilateral Clustered Single-Factor Model).

**Table 1.** Short description of the confirmatory factor models.

Name of the model	Description	Equivalent preregistered model
Clustered Indicator-Only Model	The average marker values of 10 functional clusters of 52 tracts are included as independent manifest variables and no general factor is modeled.	Indicator-Only Model
Clustered Single-Factor Model	A general factor loads onto the 10 functional cluster-average indicators (see Fig. 2A).	Single-Factor Model
Clustered Bifactor Model	A general factor is included as a primary dimension and loads onto the 10 functional cluster-average indicators. For the secondary dimension, two orthogonal latent factors capture the common variance for functional clusters of association and functional clusters of projection fibers, respectively (see Fig. 3A).	Bifactor Model
Clustered Hemisphere-Bifactor Model	A general factor is included as a primary dimension and loads onto the 10 functional cluster-average indicators. For the secondary dimension, two orthogonal latent factors capture the common variance of tract clusters in the left and tract clusters in the right hemisphere (see Fig. 4A).	No preregistered equivalent
Clustered Hierarchical Model	The general factor loads onto a latent variable association fibers and a latent variable projection fibers, which in turn load onto the 10 functional cluster-average indicators (see Fig. 3B).	Hierarchical Model
Clustered Hierarchical Hemisphere-Bifactor Model	A general factor is included as a primary dimension and loads onto two latent factors for association and projection fibers, which in turn load onto the 10 functional cluster-average indicators. For the secondary dimension, two orthogonal latent factors capture the common variance of tract clusters in the left and tract clusters in the right hemisphere (see Fig. 4B).	No preregistered equivalent
Bilateral Clustered Indicator-Only Model	The average marker values of 10 functional clusters of 52 tracts are included as independent manifest variables and no general factor is modeled. Bilateral cluster latent variables capture the shared variance of the same functional cluster across both hemispheres.	Indicator-Only Model with tract-specific latent variables
Bilateral Clustered Single-Factor Model	A general factor loads onto the bilateral cluster latent variables that capture the shared variance of the same functional cluster across both hemispheres (see Fig. 2B).	Single-Factor Model with tract-specific latent variables
Bilateral Clustered Bifactor Model	A general factor is included as a primary dimension and loads onto bilateral cluster latent variables capturing the shared variance of the same functional cluster across both hemispheres. For the secondary dimension, one latent factor captures the common variance of the bilateral functional clusters of association fibers. No bifactor for bilateral clusters of projection fibers is included as there is only one bilateral cluster of projection fibers.	Bifactor Model with tract-specific latent variables
Bilateral Clustered Hemisphere-Bifactor Model (BiClusHem-BiF Model)	A general factor is included as a primary dimension and loads onto bilateral cluster latent variables capturing the shared variance of the same functional cluster across both hemispheres. For the secondary dimension, two orthogonal latent factors capture the common variance for functional clusters in the left and right hemispheres of the brain.	No preregistered equivalent
Bilateral Clustered Hierarchical Model	A general factor loads onto a latent variable association fibers and the bilateral cluster latent variable of projection fibers. The latent variable association fibers, in turn, load onto the bilateral cluster latent variables capturing the shared variance of the same functional clusters of association fibers across both hemispheres.	Hierarchical Model with tract-specific latent variables
Bilateral Clustered Hierarchical Hemisphere-Bifactor Model (HiBiClusHem-BiF Model)	A general factor is included as a primary dimension and loads onto latent variable association fibers and the bilateral cluster latent variable of projection fibers. The latent variable association fibers, in turn, load onto the bilateral cluster latent variables capturing the shared variance of the same functional clusters of association fibers across both hemispheres. For the secondary dimension, two orthogonal latent factors capture the common variance for functional clusters in the left and right hemispheres of the brain.	No preregistered equivalent

*Note*: Each model is separately specified for the three markers.

**Fig. 4.**
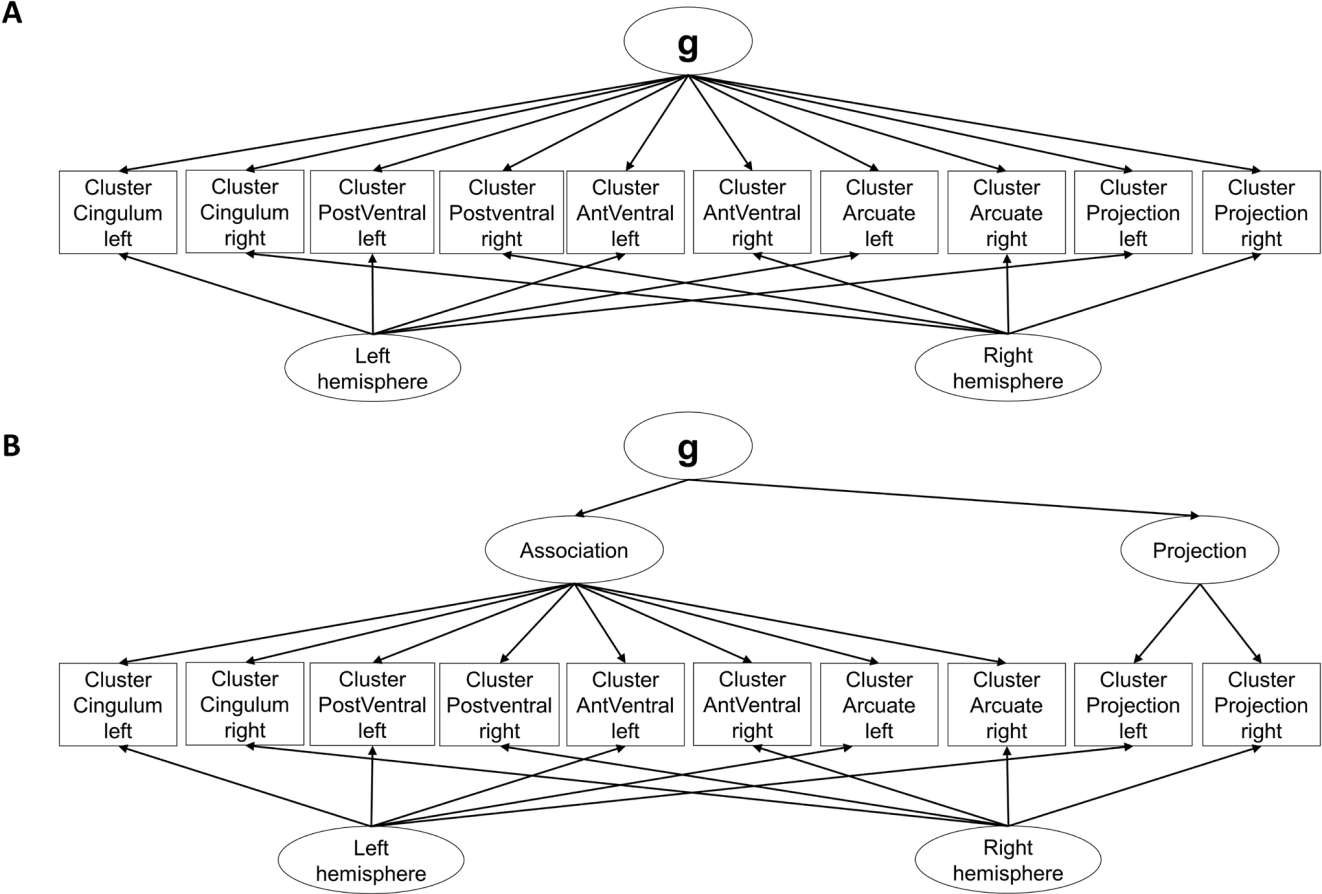
Exemplary measurement models with hemisphere bifactors. *Note*: In the Clustered Hemisphere-Bifactor Model, a general factor is included as a primary dimension and two latent variables (left and right hemisphere) are included as a secondary dimension (A). The Clustered Hierarchical Hemisphere-Bifactor Model is similar to (A) but two latent factors for association and projection fibers are included as an additional hierarchy level (B). Residual errors and the data source regressor are not depicted for simplification.

Additionally, we developed exploratory models in which the two brain hemispheres were represented as orthogonal bifactors (Fig. 4A, B). While these models were not preregistered, we included them based on data-driven observations: tract-wise associations were notably stronger within hemispheres than between them.

#### 2.5.3 Measurement model fluid intelligence

To construct the latent variable fluid intelligence, we performed an odd–even item split of the Raven’s Progressive Matrices or Advanced Progressive Matrices, respectively. We included manifest indicators representing the proportion of correctly solved odd items and the proportion of correctly solved even items. To ensure local identifiability, the factor loadings of the latent variable on both indicators were constrained to be equal. IQ values were not used to model the latent variable fluid intelligence but only computed for the sample descriptions.

### 2.6 Exploratory analyses

As specified in our preregistration, we repeated our analysis controlling for age to explore the robustness of our results. To do so, we included a regression of all latent variables on age in all models and repeated the systematic model comparison.

## 3 Results

### 3.1 Descriptive results

**Fig. 5.**
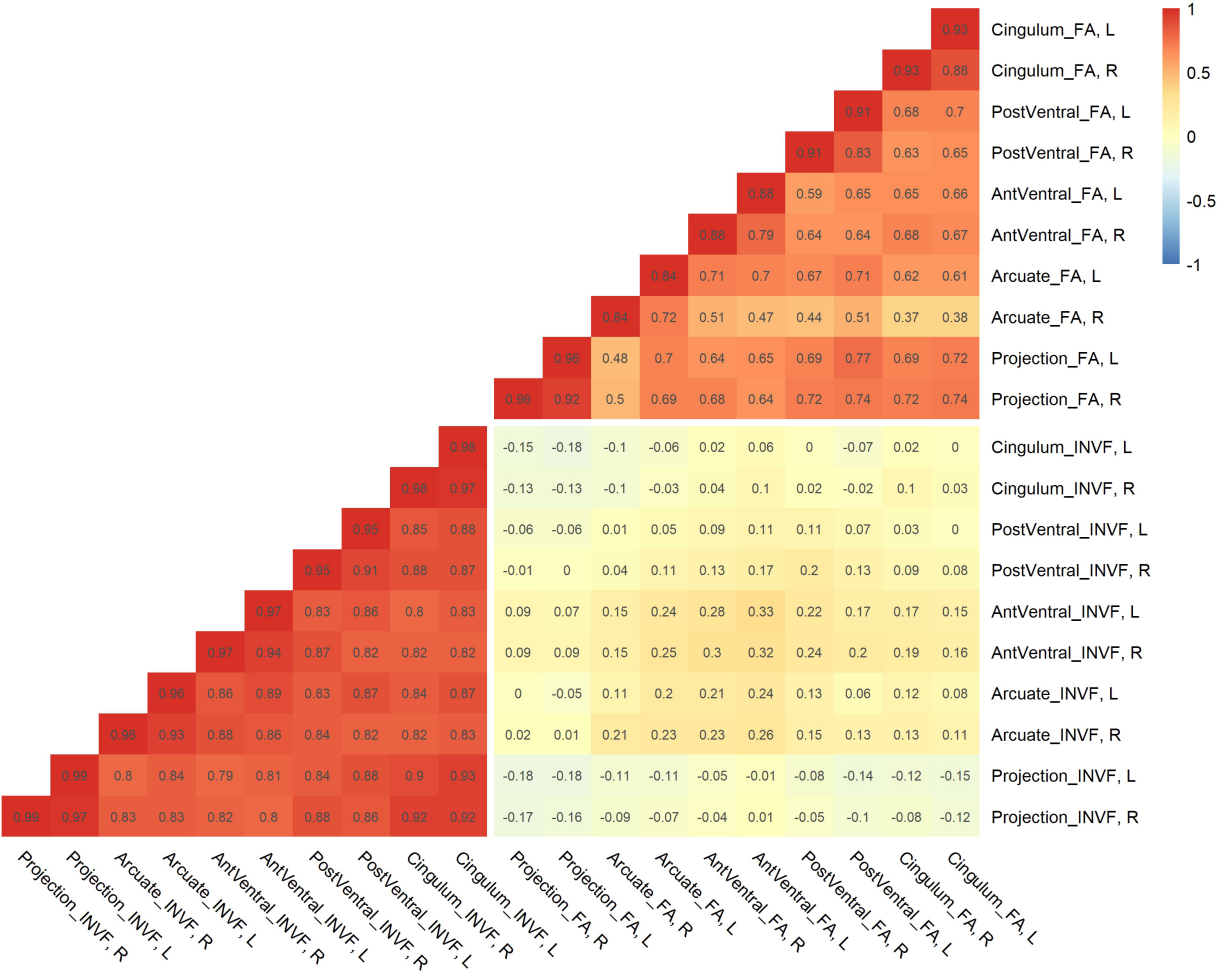
Full partial correlation matrix of the MRI-derived indicator variables FA and INVF for the combined data. *Note*: Partial Pearson correlations controlled for data source between cluster averages of the FA and INVF markers in the combined sample. Split-half reliabilities of the left and right clusters are presented on the diagonal. AntVentral = anterior ventral system, PostVentral = posterior ventral system, FA = fractional anisotropy, INVF = intraneurite volume fraction, R = right hemisphere, L = left hemisphere.

The full partial correlation table of the manifest indicator variables for the FA and INVF markers in the combined data controlled for data source is shown in Figure 5. The cluster averages for FA and INVF in the combined data yielded good to excellent reliabilities computed as the split-half reliability of the left and right cluster, 0.84 ≤ *r_split–half_* ≤ 0.99.

**Fig. 6.**
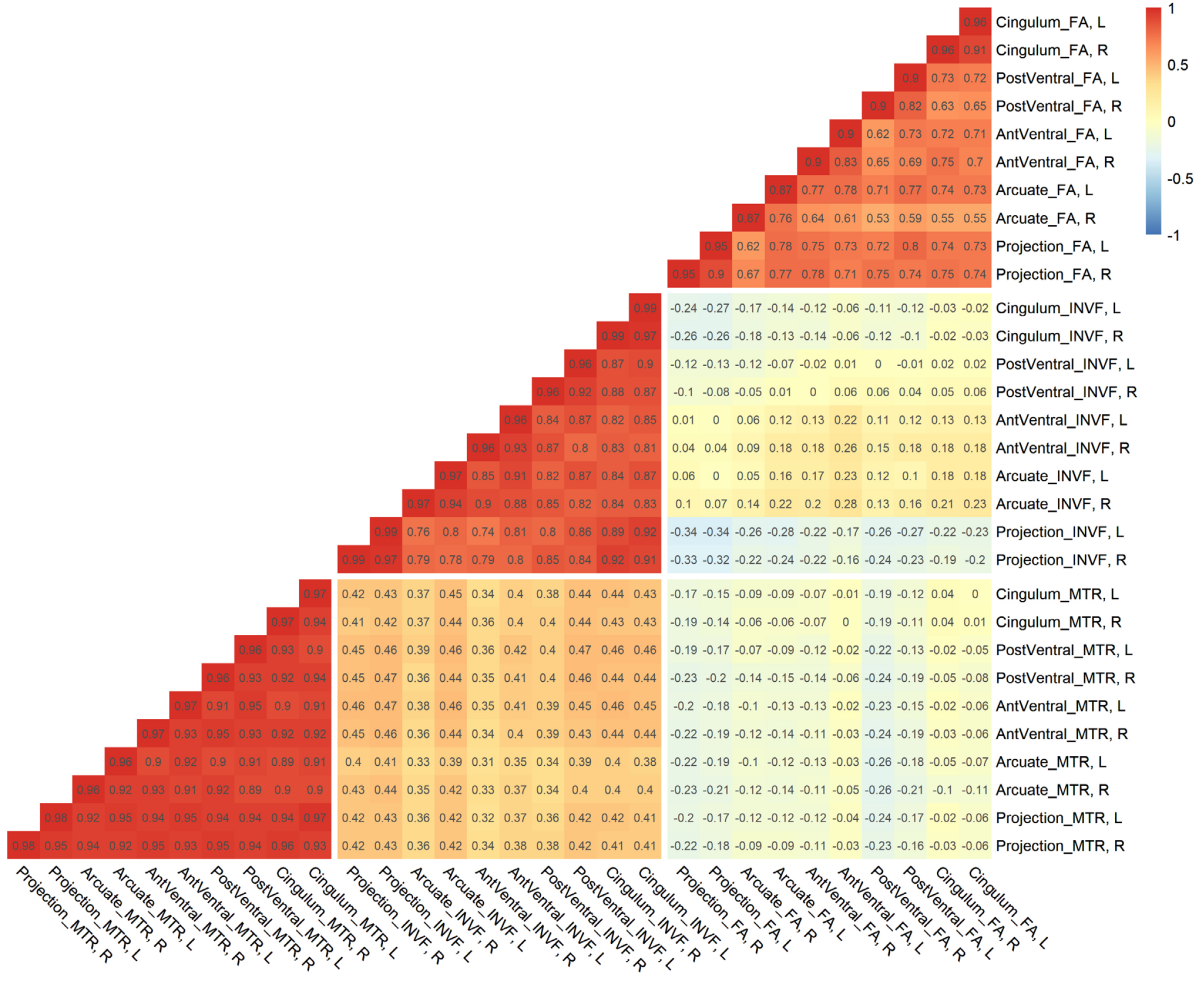
Full correlation matrix of the MRI-derived indicator variables FA, INVF, and MTR for the Dortmund data. *Note*: Pearson correlations between cluster averages of the FA, INVF, and MTR markers in the Dortmund data. Split-half reliabilities of the left and right clusters are presented on the diagonal. AntVentral = anterior ventral system, PostVentral = posterior ventral system, FA = fractional anisotropy, INVF = intraneurite volume fraction, MTR = magnetization transfer ratio, R = right hemisphere, L = left hemisphere.

We computed the full correlation table of the manifest indicator variables for the FA, INVF, and MTR markers in the Dortmund data only because MTR was only available in this data set (Fig. 6). Similarly, the split-half reliabilities of the cluster averages for FA, INVF, and MTR in the Dortmund data proved to be good to excellent, 0.87 ≤ *r_split–half_* ≤ 0.98.

### 3.2 White matter microstructure measurement models

We consistently identified the Bilateral Clustered Hemisphere-Bifactor Model and the Bilateral Clustered Hierarchical Hemisphere-Bifactor Model across all markers as winning measurement models (hereafter: BiClusHem-BiF Model and HiBiClusHem-BiF Model). Both models extract a general factor representing the respective white matter microstructure marker as a general property of the brain. Additionally, both models include the left and right tract clusters as bilateral cluster factors, capturing the common variance across hemispheres. They also both specifically model the brain hemisphere as bifactors. The HiBiClusHem-BiF Model also introduces an additional hierarchy level, where a latent variable captures the common variance across all tract clusters of association fibers. Below, we summarize the findings for all markers individually.

**Fig. 7.**
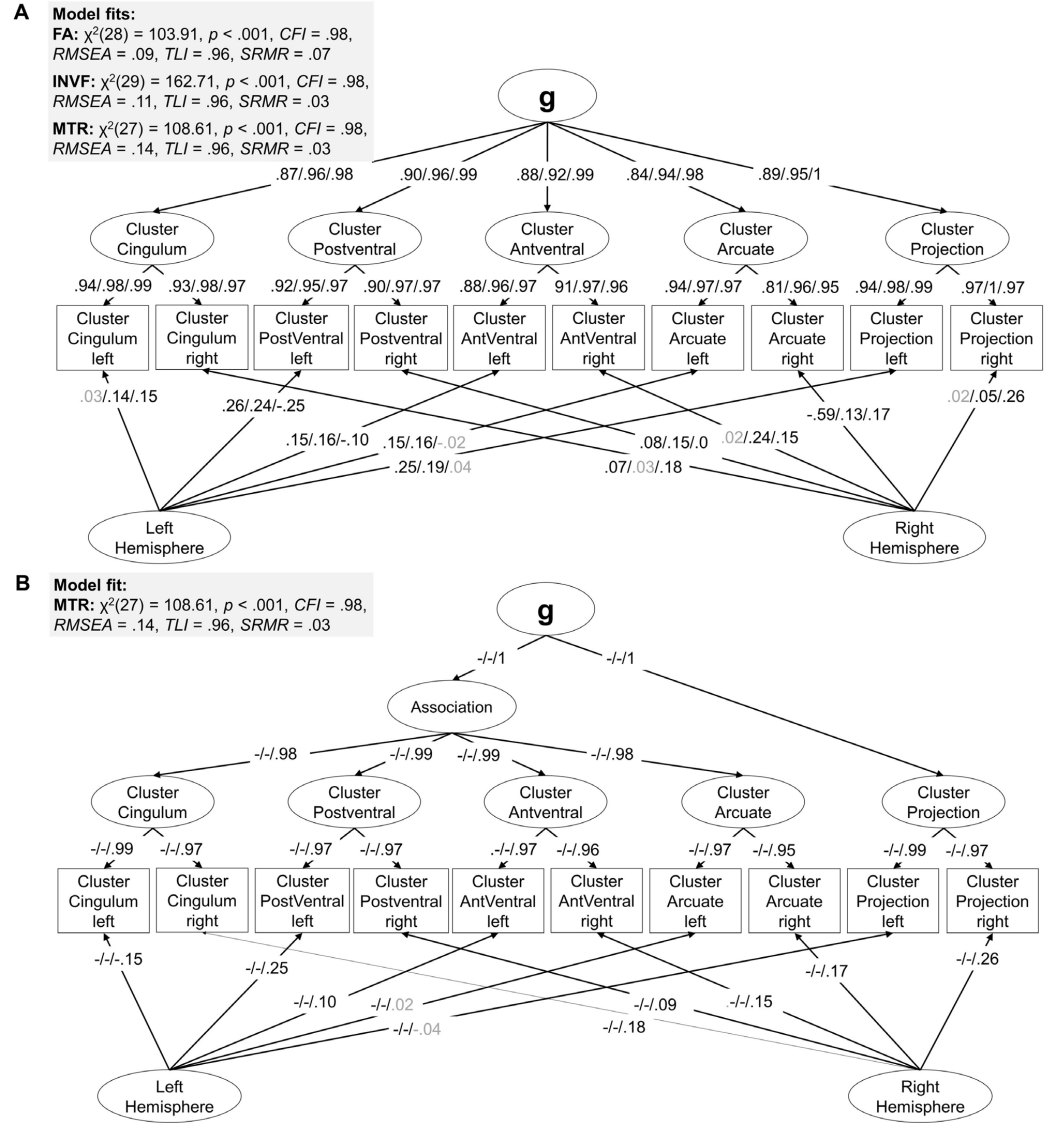
Winning models. *Note*: Standardized factor loadings for the BiClusHem-BiF Model are shown for FA/INVF/MTR (A). Standardized factor loadings for the HiBiClusHem-BiF Model are shown for -/-/MTR. We do not report factor loadings of the FA and INVF markers for this model as it was only selected for MTR (B). Residual errors and the data source regressor are not shown for simplification. Insignificant factor loadings are grayed out.

The systematic model comparison identified the BiClusHem-BiF Model as the best measurement model representing white matter integrity (FA marker, Fig. 7A), *AIC* = 6473.08, *AIC_weight_* = 0.73, followed by the HiBiClusHem-BiF Model, *AIC* = 6475.08, *AIC_weight_* = 0.27. The model fit indices of all competing models are presented in Supplementary Table S8.

For neurite density (the INVF marker), the BiClusHem-BiF Model (Fig. 7A) was also decisively selected as the winning model, *AIC* = 3004.44, *AIC_weight_* = 1. The model fits of all competing INVF models are summarized in Supplementary Table S9. The model showed good fit on CFI, TLI, and SRMR. RMSEA exceeded conventional thresholds; however, modification indices revealed no anatomically or theoretically justified adjustments. We retained the model as specified for subsequent analyses.

We ran the myelin content (MTR marker) model comparison on the Dortmund data and not on the combined data as the MTR marker was only available for this group. We identified both the BiClusHem-BiF Model (Fig. 7A) and the HiBiClusHem-BiF Model (Fig. 7B) as the winning measurement models, both *AIC* = −1628.20, *AIC_weight_* = 0.50 (see Supplementary Table S10 for all model fits). Similar to the findings for the INVF marker, the RMSEA indicated an unacceptable model fit, contrasting with all other model fit indices. Because the suggested modifications could not be justified by anatomical or theoretical considerations, we chose not to adjust the model to avoid overfitting.

After identifying a best-fitting structure for each microstructural marker, we then built a joint white matter microstructure model to investigate the latent associations between white matter integrity, neurite density, and myelin content.

### 3.3 The latent relationship between white matter integrity, neurite density, and myelin content

We constructed two versions of joint models from the previously chosen winning measurement models. In the first version, we included the BiClusHem-BiF Models for all three markers. The second version used the HiBiClusHem-BiF Model for MTR. For both versions, we tested multi-factor solutions against hierarchical solutions. Model fit indices of all competing models are presented in Supplementary Table S11. The two hierarchical models extracting a higher-order general factor from the factors FA, INVF, and MTR produced the warning that the variance–covariance matrix of the estimated parameters and the covariance matrix of latent variables were not positive definite. Consequently, we excluded these models from model comparison.

The winning joint white matter model was the combined model with freely correlating latent factors FA, INVF, and MTR, and identical measurement models for all markers, *AIC* = 10303.95, *AIC_weight_* = 0.57. This model revealed that individuals with a higher neurite density (general factor INVF) displayed a significantly higher myelin content (general factor MTR) when measured at a general level, *r* = 0.46, *p* < .001, 95 % CI: [0.33 0.59]. There was no significant association between the general factors FA and INVF, *r* = 0.03, *p* = .640, 95 % CI: [-0.09 0.14], or the general factors FA and MTR, *r* = -0.11, *p* = .181, 95 % CI: [-0.26 0.05].

Next, we included five orthogonal method factors to account for commonalities of the FA, INVF, and MTR measures extracted from the same functional cluster. The method factors loaded onto the bilateral cluster variables of the five functional systems. However, this model yielded the warning that the variance–covariance matrix of the estimated parameters was not positive definite, leading us to reject it, retaining the model without these methods factors.

Having established measurement models for each marker and a joint white matter model relating the three markers, we now turn to the central question: How do these white matter properties relate to fluid intelligence?

### 3.4 Including fluid intelligence in the joint model

**Fig. 8.**
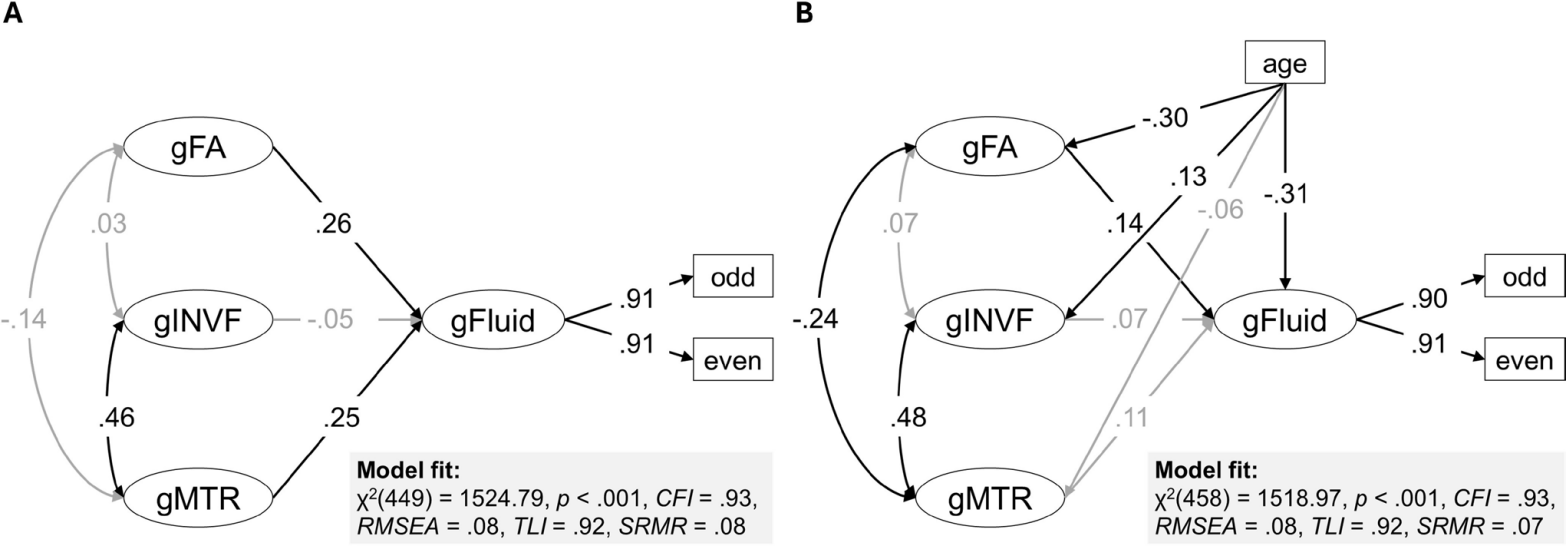
White matter microstructure and fluid intelligence. *Note*: The full structural models depict the latent relationships (standardized loadings) between white matter microstructure and fluid intelligence (A) and the full structural model with age included as a covariate (B). Residual errors and the data source regressor are not shown for simplification. Insignificant paths are grayed out. gFA = general factor fractional anisotropy, gINVF = general factor intraneurite volume fraction, gMTR = general factor magnetization transfer ratio, gWM = general factor white matter microstructure, gFluid = general factor fluid intelligence.

To answer this question, we included fluid intelligence as a latent dependent variable in the previously selected joint white matter model (see Fig. 8A). Fluid intelligence was significantly related to white matter integrity (general factor FA), β = 0.26, *p* < .001, 95 % CI: [0.14 0.41], and myelin content (general factor MTR), β = 0.25, *p* = .017, 95 % CI: [0.05 0.48]. Neurite density (general factor INVF) showed no significant relationship with fluid intelligence, β = -0.05, *p* = .518, 95 % CI: [-0.21 0.10].

### 3.5 Controlling for the influence of age

Given the substantial age heterogeneity in the combined sample (range: 18–74 years) and the well-established age-related declines in both white matter microstructural markers and fluid intelligence, we repeated all analyses while controlling for age to assess the robustness of our findings. Specifically, we residualized age with respect to data set source, and included the resulting residuals in all measurement models during model comparison as well as in all subsequent joint models.

Consistent with the analysis without age as a covariate, we identified the BiClusHem-BiF Model for the FA marker, *AIC* = 6412.40, *AIC_weight_* = 1. For INVF, we again identified the BiClusHem-BiF Model, *AIC* = 2950.02, *AIC_weight_* = 0.88, followed by the HiBiClusHem-BiF Model, *AIC* = 2954.02, *AIC_weight_* = 0.12. Finally, for MTR, we again identified both the BiClusHem-BiF Model, *AIC* = −1624.83, *AIC_weight_* = 0.50, and the HiBiClusHem-BiF Model, *AIC* = −1624.87, *AIC_weight_* = 0.50 (see Supplementary Tables S12, S13, and S14 for model fits).

For consistency with the joint white matter model without age as covariate, we constructed the age-controlled joint white matter model using the BiClusHem-BiF Model for all markers, yielding acceptable model fit according to the CLI, TLI, and SRMS but slightly above cutoff RMSEA. Finally, we included a dependent latent variable fluid intelligence in the joint model (see Fig. 8B).

After controlling for age, we still observed a significant effect of the general factor FA on fluid intelligence, β = 0.14, *p* = .043, 95 % CI: [0.01 0.28]. There was no longer a significant effect of the general factor MTR on fluid intelligence, β = 0.11, *p* = .328, 95 % CI: [-0.12 0.35]. Again, the general factors INVF and fluid intelligence were unrelated, β = 0.07, *p* = .371, 95 % CI: [-0.09 0.24]. The influence of age on all latent variables and their relationships is depicted in Figure 8B.

## 4 Discussion

In this study, we characterized the factor structure of several MRI-derived white matter microstructure markers and investigated their potential role as biological bases of fluid intelligence. Hierarchical models with bifactors for the hemispheres proved optimal for all markers independent of age. We found that a higher white matter integrity and myelin content were significantly associated with higher fluid intelligence. When controlling for age, the association between white matter integrity and fluid intelligence remained significant, while the association between myelin content and fluid intelligence was no longer significant. This finding partially supports our hypothesis stating that individuals with higher fluid intelligence would exhibit greater white matter integrity, neurite density, and myelin content. Below, we interpret and discuss our findings and their implications with respect to the anatomical organization of white matter tracts and their microstructural properties as well as with respect to their impact on fluid intelligence.

### 4.1 Hierarchical measurement models with hemisphere bifactors win the model comparison

We either identified the BiClusHem-BiF Model or the HiBiClusHem-BiF Model for all three markers independent of age. Both models share important features such as the extraction of a general factor, which is consistent with previous accounts on the white matter microstructure factor structure (Cox et al., 2016; Penke et al., 2012). The general factor implies that white matter integrity, neurite density, and myelin content constitute general properties of the brain, and that despite anatomical and functional differences of the tract clusters, there are likely shared underlying properties such as common genetic factors that influence all clusters in a similar matter. Genetic accounts support this notion, showing that microstructural properties of white matter are heritable across tracts as demonstrated for white matter integrity (the FA marker, Chiang et al., 2009; Kochunov et al., 2015; Koenis et al., 2015), neurite density (the INVF marker, Kochunov et al., 2016), myelin content (the MTR marker, Brouwer et al., 2010), and total white matter volume (Posthuma et al., 2002). Importantly, the general factors of white matter integrity and myelin content showed a robust association with fluid intelligence, supporting its interpretation as a cognitively relevant dimension of brain-wide white matter properties rather than a purely statistical summary of covariance. These findings align with frameworks in which individual differences in fluid intelligence reflect widely distributed neural properties rather than isolated regional features. If white matter microstructure and myelin content contribute to the efficiency of large-scale neural communication, then a general factor capturing covariance across major tracts provides a plausible, empirically supported candidate mechanism linking global white matter properties to cognitive functioning.

Additionally, both winning measurement models include cluster-specific latent variables for the cingulum, posterior ventral, anterior ventral, arcuate, and projection fiber clusters, capturing the shared variance across the left and right hemispheres. This suggests that white matter within a specific functional system tends to be homogeneous across hemispheres in its integrity, density, and myelination. This observation may be driven by often higher correlations of microstructural properties in homologous tracts across hemispheres compared with non-homologous tracts within the same or different hemispheres as previously demonstrated for white matter integrity (Li et al., 2012; Wahl et al., 2010). This notion is also supported by previous findings (Laukka et al., 2013; Lövdén et al., 2013), which demonstrated good fit of models with tract-specific latent variables modeling white matter integrity in homologous tracts across hemispheres.

Also, both models include hemispheric bifactors that capture additional variance specific to the left or right hemisphere. This suggests that despite sharing microstructural properties across hemispheres (as captured by the latent cluster-specific variables), some aspects of white matter microstructure remain different in the two hemispheres. Hemispheric asymmetries in microstructural organization or expression, such as higher or more variable microstructure marker values in one hemisphere over the other, have been demonstrated for white matter integrity in the past (Eluvathingal et al., 2007; Thiebaut de Schotten et al., 2011). Hemispheric asymmetries may underlie a specialization of specific white matter tracts in certain tasks known to show lateralization effects such as leftward dominance for language (see Josse & Tzourio-Mazoyer (2004) for a review) or cognitive functioning (Cabeza, 2002; Hennessee et al., 2022; Reuter-Lorenz & Cappell, 2008) that are likely mapped by the hemispheric bifactors in this study’s latent models.

For the MTR marker only, we also selected the HiBiClusHem-BiF Model. In this model, a latent variable capturing the shared variance of all association fibers tract clusters is included as an additional hierarchy level. This latent variable highlights the anatomical distinction between association and projection fibers: the myelin content in two association tracts is more similar to each other compared with the myelin content in a projection tract.

Together, our identified measurement models suggest that white matter and its microstructural properties are hierarchically organized, with tracts clustering into functionally meaningful groups that share microstructural properties also across hemispheres. By extracting a general factor, we showed that properties of white matter microstructure form a global component influencing all tract clusters. Hemispheric differences in microstructure suggest that certain brain functions may be more dependent on specific white matter characteristics in one hemisphere over the other. The measurement models reinforce the idea that differences in the integrity, the neurite density, and the myelin content are not just localized to individual tracts, but also rather follow a hierarchical organization.

### 4.2 Markers of white matter microstructure are differentially related with each other

We expected positive latent associations between the three general factors of the white matter microstructure markers, because neurite density and myelin content are considered key drivers of white matter integrity, with high values in both favoring greater integrity (Beaulieu, 2010). Also, the INVF marker has previously been shown to be positively associated with white matter integrity across the brain (Zhang et al., 2012) and in individual tracts (e.g., Friedrich et al., 2020). However, we observed an unexpected pattern: While there was a positive latent association between the general factors INVF and MTR (*r* = .46 and *r* = .48 when controlled for age), there were no significant latent correlations between the general factors FA and INVF or FA and MTR. When controlling for age, there was even a significant negative latent association between the general factors FA and MTR (*r* = -.24), indicating that individuals with higher white matter integrity exhibited lower myelin content.

To further explore the negative association between the general factors FA and MTR, we examined the pairwise correlations between FA and MTR averages across all tract clusters and across individual tracts in the Dortmund data. At the cluster level, we observed the strongest negative associations between FA in bilateral posterior ventral and projection fiber clusters with MTR in all other clusters (see Supplementary Figure S7). At the tract level, we saw that the negative association between FA and MTR was primarily driven by the bilateral posterior and superior segments of the corticostriatal tracts, the inferior fronto-occipital fasciculus, the occipital radiation, the anterior, posterior, and superior thalamic radiations, and the uncinate fasciculus (see Supplementary Figure S9). Participants with high FA values in these tracts tended to show lower MTR values in most other tracts.

We also explored the zero correlation between the general factors FA and INVF by examining the partial pairwise correlation between FA and INVF controlled for data source in the combined data. At the cluster level, INVF in bilateral projection fiber clusters and, to a lesser extent, in bilateral cingulum clusters showed negative correlations with FA across all clusters while all other clusters tended to be positively associated, highlighting a distinct pattern in the projection and cingulum systems (see Supplementary Figure S8). At the individual tract level, we observed that the zero association between FA and INVF was primarily driven by negative associations in the bilateral parahippocampal and parahippocampal parietal segments of the cingulum, corticobulbar and corticospinal tracts, fornix, and anterior thalamic radiations (see Supplementary Figure S10). Participants with high INVF values in these tracts tended to show lower FA values in most other tracts.

One possible explanation for this divergence is a known limitation of the FA metric: it captures only the primary diffusion direction and fails to resolve fiber crossing and dispersion (Wiegell et al., 2000). In brain regions where multiple fiber bundles intersect or fan out, FA values may be artificially lowered due to reduced directional coherence, even when myelin content and neurite density (and thus MTR and INVF) remain high. That regions of crossing or fanning fibers can impact DTI-derived metrics such as the FA is a known shortcoming of the technique and has been demonstrated for the corticospinal tract as an example (Lee et al., 2015).

To test this possible explanation, we inspected the pairwise correlations between FA and MTR in the Dortmund data on an individual tract level for tracts that are more strongly versus less strongly affected by fiber crossing and fanning (see Supplementary Figure S11). Similarly, we assessed the pairwise partial correlation between FA and INVF in the combined data controlled for data source in tracts that are more strongly versus less strongly affected by fiber crossing and fanning (see Supplementary Figure S12). To quantify crossing and fanning, we used the orientation dispersion index (ODI) map that is estimated alongside the INVF map by the NODDI model. ODI in white matter is a marker for axonal bending, crossing, or fanning (Zhang et al., 2012). We calculated average ODI values for all 52 tracts and identified the 10 tracts with the highest average ODI values as well as the 10 tracts with the lowest average ODI values. We observed lower (partial) correlations in most high ODI tracts as compared with low ODI tracts, supporting the idea that FA values may be artificially lowered in areas of reduced directional coherence, even when MTR and INVF values remain high.

### 4.3 White matter integrity and myelin content are significantly associated with fluid intelligence

The findings from this study provide further evidence for white matter integrity (FA) as a structural basis of fluid intelligence independent of age, aligning with prior research (Booth et al., 2013; Ferrer et al., 2013; Fuhrmann et al., 2020; Genç et al., 2018; Kievit et al., 2016; Penke et al., 2012; Stammen et al., 2023; Tamnes et al., 2010; Wendelken et al., 2017). In addition, our study adds specificity by incorporating two less commonly investigated markers of white matter microstructure and demonstrating that higher myelin content but not a higher neurite density is significantly related to higher fluid intelligence. When participants’ age was included as a covariate, this association no longer reached significance. However, we argue that this does not render the relationship artifactual. Rather, the covariate age may reflect genuine age-related changes in myelin content, which in turn may contribute to age-related variation in fluid intelligence. In this sense, our finding aligns with earlier work showing that a general MTR factor is significantly related to general intelligence within an SEM framework (Penke et al., 2012). Additionally, prior research has shown that intensive cognitive training can induce white matter plasticity, potentially through increased myelination, while also improving cognitive performance across domains such as working memory, episodic memory, and perceptual speed (Lövdén et al., 2010). The authors observed a significant intervention-related decrease in radial but not in axial diffusivity, a pattern they link to increased myelination (see also Alexander et al., 2007), reinforcing myelin as a potential neural basis of fluid intelligence.

Integrating our findings into the neurocognitive process model proposed by Schubert and Frischkorn (2020), it appears that higher myelin content enhances white matter tract integrity and facilitates faster information processing, given the relatively direct relationship between myelin and conduction velocity along axons. In turn, the speed of information processing likely mediates the relationship between white matter microstructure and intelligence (e.g., Ferrer et al., 2013; Fuhrmann et al., 2020; Kievit et al., 2016). Future research should explicitly model processing speed as an intermediate variable, in order to empirically test the neurocognitive process model and clarify the mechanistic pathways linking myelination, mental speed, and cognitive ability.

### 4.4 Strengths and limitations

An important strength of this study is the inclusion of a second independent and large data set beyond the preregistered Dortmund sample. This allowed us to assess the white matter microstructure factor structure as well as the latent correlation between the markers and fluid intelligence in a larger sample, thus emphasizing the robustness of our findings.

Another strength of this study is our multiple marker approach. By including two less frequently investigated indices of white matter microstructure, we extended prior work that has primarily relied on FA. This contributes to a more nuanced characterization of white matter architecture and its role in fluid intelligence.

However, some limitations should be acknowledged. In this study, fluid intelligence was operationalized based on a single measure in each sample (Dortmund sample: Raven’s Progressive Matrices 2; Mainz sample: Advanced Progressive Matrices). Although data from the Berlin Intelligence Structure Test were available in the Mainz sample, we decided against including these scores to ensure consistency across data sources. A potential limitation of this approach is that assessing fluid intelligence using a single test may limit measurement precision and, consequently, affect associations with markers of brain white matter microstructure, similar to what has been reported for the link between general intelligence and brain volume (Gignac & Bates, 2017). Nevertheless, we argue that this concern is mitigated by the fact that Raven’s Progressive Matrices are widely regarded as being among the best single indicators of general intelligence (Snow & Lohman, 1984), but we acknowledge that fluid intelligence would be measured more comprehensively in broader test batteries.

A downside of including two independent data sets is the inconsistency in the assessment of fluid intelligence across them. Although both studies used matrix reasoning tests, the test conditions varied. In the Dortmund sample, participants completed the Raven test under a 45-minute time limit, whereas in the Mainz sample, the Advanced Progressive Matrices were administered without a time constraint. Although the 45-minute limit aligns with the test manual, it may have introduced speeding in the Dortmund sample (Gonthier, 2023), altering the cognitive demands of the task. Earlier research has shown that speeded tests of fluid intelligence become statistically indistinguishable from working memory capacity when only half of the recommended time to complete the test is given (Chuderski, 2013). In the Mainz sample, the Advanced Progressive Matrices were administered without the 40-minute time limit recommended in the manual. Although the standardization of IQ scores is based on the timed version, the manual explicitly permits untimed administration, noting only that norm-based IQ values may not be applicable in such cases (Heller et al., 1998). Since our analysis relied on percentage correct scores rather than norm-referenced IQ scores, the absence of a time limit is unlikely to have affected the validity of the test in this context. Moreover, unspeeded administration ensures that the test functions as a measure of reasoning ability (i.e., a power test) rather than processing speed, which may be particularly advantageous depending on the research focus. Nevertheless, the difference in intelligence test administration (timed vs. untimed) across samples potentially introduces variations across the two included data sets that may affect the relationship with the white matter microstructure markers.

Additionally, differences in the MRI scanner hardware and diffusion imaging protocols between sites present an additional source of variance that could influence microstructural estimates. We explicitly decided against a data harmonization strategy such as ComBat (Fortin et al., 2017) to remove site effects from the neuroimaging data because data harmonization can induce correlations between originally independent subjects (Hu et al., 2023; Li et al., 2023). This in turn would change the underlying covariance structure of the data affecting the structural equation modeling that relies on the covariance matrix. Instead, we have combined the data by first *z*-standardizing within each sample and then across the two samples to minimize site effects.

Another limitation concerns the high RMSEA values that we sometimes observed for the individual markers measurement models and that suggested unacceptable model fit, while all other fit indices (CFI, TLI, and SRMR) consistently indicated acceptable to good fit. We deliberately chose not to modify our models based on modification indices to avoid overfitting and because the suggested changes lacked theoretical or anatomical justification. We argue that the elevated RMSEA values should be interpreted with caution. The RMSEA may over-penalize model complexity, especially in the context of moderate degrees of freedom (all selected measurement models had *df* ≤ 34). Prior research has shown that the RMSEA tends to be inflated in models with small sample sizes and low degrees of freedom, even when the model is correctly specified (Kenny et al., 2015). All of our measurement models include multiple latent variables and impose equality constraints to ensure local identifiability. These constraints increase the structural rigidity of the models, and as a result, even minor global misfits are amplified, which likely contributes to the inflation of the RMSEA despite overall good model performance based on other indices.

Finally, the reliance on MRI-derived proxies without direct histological or additional imaging validation limits the interpretability of the underlying biology. The MTR marker, for instance, has been criticized because a change in MTR is not necessarily attributable to a change in myelin (MacKay & Laule, 2016) but could be caused by inflammation or edema (Vavasour et al., 2011). Similarly, evidence from a murine model of spinal cord injury suggests that spontaneous remyelination may not be captured by MTR, but rather by the myelin water fraction (MWF), which is thought to be a more specific marker of myelin (McCreary et al., 2009). Unfortunately, MWF measures were not available in the present data sets, necessitating the use of MTR as an indicator of myelin content.

### 4.5 Conclusion

In this study, we derived consistent measurement models of three white matter microstructure markers in a large sample and independent of age. Our findings demonstrate that greater white matter integrity (even when controlled for age) and myelin content (not significant when controlled for age) are associated with higher fluid intelligence. From the perspective of the neurocognitive process model proposed by Schubert and Frischkorn (2020), our findings support the hypothesis that increased myelin content enhances white matter tract integrity and facilitates faster information processing by increasing conduction velocity along axons.

Overall, this study advances a methodological framework for integrating MRI-derived markers into individual differences research via structural equation modeling while simultaneously offering novel insights into the neurobiological foundations of fluid intelligence. The measurement models developed in this study provide a robust foundation for future longitudinal research on white matter microstructure and cognitive development or aging. For example, the longitudinal design of the Dortmund Vital Study allows future research, to validate the present findings in a longitudinal framework and to investigate the white matter microstructure factor structure across the lifespan. By establishing reliable latent structures for FA, INVF, and MTR, these models enable the assessment of within-person change in microstructural properties over time, while minimizing measurement error and isolating tract-specific versus global effects. Importantly, the inclusion of hemisphere- and cluster-specific latent variables allows researchers to explore differential rates of change across regions and fiber types. This opens the door for investigating whether intra-individual trajectories of white matter integrity, neurite density, or myelination predict subsequent changes in cognitive ability, or vice versa, thereby helping to clarify the directionality and causality of the white matter–intelligence relationship across the lifespan.

## Supplementary Material

Supplementary Material

## Data Availability

The research data and analysis code are available on the OSF (https://osf.io/nhxcg/files).
