# Supplementary material for: The latent organization of white matter microstructure and its relation to fluid intelligence
Source: Imaging Neurosci (Camb). 2026 Mar 19;4:IMAG.a.1167. doi: 10.1162/IMAG.a.1167 (PMC13003808; doi:10.1162/IMAG.a.1167)
Supplement: Supplementary Material [file IMAG.a.1167_supp.pdf]

Supplementary Material

The Psychometric Structure of White Matter and its Relation to Fluid Intelligence

Henrike M. Jungeblut[a], Erhan Genç[b], Michael Burke[b], Patrick D. Gajewski[b], Stephan Getzmann[b], Edmund Wascher[b,c], and & Anna-Lena Schubert[a]

[a] Department of Psychology at Johannes Gutenberg University
Wallstraße 3
55122 Mainz
Germany

[b] Leibniz Research Centre for Working Environment and Human Factors
at Dortmund University
Ardeystraße 67
44139 Dortmund Germany

[c] German Center for Mental Health (DZPG) partner site Bochum/Marburg
Massenbergstraße 9-13
44787 Bochum
Germany

Supplementary Table 1

Short description of the preregistered models

Name of the model	Description
Indicator-Only Model	The 52 tract-average marker values are included as independent manifest variables and no general factor is modeled.
Single-Factor Model	A general factor loads onto all 52 tract-average indicators.
Bifactor Model	A general factor is included as a primary dimension and two latent variables (association and projection fibers) are included as a secondary dimension.
Hierarchical Model	The general factor loads onto a latent variable association fibers and a latent variable projection fibers, which in turn load onto the manifest indicators.
Multi-Factor Model	The latent variable for association fibers from the Hierarchical and Biactor Model is further decomposed into four latent variables representing four systems of association fibers. These systems are the cingulum system, the posterior ventral system, the anterior ventral system, and the arcuate system. In addition to these four latent variables, a latent variable for projection fibers that captures the common variance of the remaining 20 projection fibers from the atlas is included. All latent variables are free to correlate with each other.
Multi-Factor/Bifactor Model	A general factor is included as a primary dimension. As a secondary dimension, the orthogonal latent variables representing the cingulum system, the posterior ventral system, the anterior ventral system, the arcuate system, and the projection fibers, are included.
Hierarchical Multi-Factor Model	A general factor that loads onto the five latent variables of the Multi-Factor Model is included.
Three-Level Multi-Factor Model	On the first hierarchy level, the model is equivalent to the Multi-Factor Model where five latent variables capture the covariance of groups of white matter tracts. On the second hierarchy level, a latent variable for association fibers loads onto the latent variables representing the cingulum, the posterior ventral, the anterior ventral, and the arcuate system. A general factor on the third hierarchy level loads onto the latent variable association fibers and the latent variable projection fibers.
Two-Level Multi-Factor Bifactor	The general factor is removed from the third hierarchy level of the Three-Level Multi-Factor Model and instead included as a primary dimension in a bifactor model. The latent variables for association and projection fibers are kept orthogonal.
Decomposed Multi-Factor Model	Modification of the Multi-Factor Model, in which the latent variable for projection fibers is further decomposed into three latent variables capturing the common variance of projection fibers innervating the brainstem and spinal cord, the striatum, and the (hypo)thalamus.
Decomposed Multi-Factor/Bifactor Model	Modification of the Multi-Factor/Bifactor Model, in which the latent variable for projection fibers is further decomposed into three latent variables capturing the common variance of projection fibers innervating the brainstem and spinal cord, the striatum, and the (hypo)thalamus.

Short description of the preregistered models (continued)

Name of the model	Description
Decomposed Hierarchical Multi-Factor Model	Modification of the Hierarchical Multi-Factor Model, in which the latent variable for projection fibers is further decomposed into three latent variables capturing the common variance of projection fibers innervating the brainstem and spinal cord, the striatum, and the (hypo)thalamus.
Decomposed Three-Level Multi-Factor Model	Modification of the Three-Level Multi-Factor Model, in which the latent variable for projection fibers is further decomposed into three latent variables capturing the common variance of projection fibers innervating the brainstem and spinal cord, the striatum, and the (hypo)thalamus.
Decomposed Two-Level Multi-Factor Bifactor	Modification of the Two-Level Multi-Factor Model, in which the latent variable for projection fibers is further decomposed into three latent variables capturing the common variance of projection fibers innervating the brainstem and spinal cord, the striatum, and the (hypo)thalamus.
Indicator-Only Model with tract-specific latent variables	Modification of the Indicator-Only Model, in which the common variance of the same tracts in the two hemispheres is captured by tract-specific latent variables.
Single-Factor Model with tract-specific latent variables	Modification of the Single-Factor Model, in which the common variance of the same tracts in the two hemispheres is captured by tract-specific latent variables.
Bifactor Model with tract-specific latent variables	Modification of the Bifactor Model, in which the common variance of the same tracts in the two hemispheres is captured by tract-specific latent variables.
Hierarchical Model with tract-specific latent variables	Modification of the Hierarchical Model, in which the common variance of the same tracts in the two hemispheres is captured by tract-specific latent variables.
Multi-Factor Model with tract-specific latent variables	Modification of the Multi-Factor Model, in which the common variance of the same tracts in the two hemispheres is captured by tract-specific latent variables.
Multi-Factor/Bifactor Model with tract-specific latent variables	Modification of the Multi-Factor/Bifactor Model, in which the common variance of the same tracts in the two hemispheres is captured by tract-specific latent variables.
Hierarchical Multi-Factor Model with tract-specific latent variables	Modification of the Hierarchical Multi-Factor Model, in which the common variance of the same tracts in the two hemispheres is captured by tract-specific latent variables.
Three-Level Multi-Factor Model with tract-specific latent variables	Modification of the Three-Level Multi-Factor Model, in which the common variance of the same tracts in the two hemispheres is captured by tract-specific latent variables.
Two-Level Multi-Factor Bifactor with tract-specific latent variables	Modification of the Two-Level Multi-Factor Model, in which the common variance of the same tracts in the two hemispheres is captured by tract-specific latent variables.

Short description of the preregistered models (continued)

**Name of the model**	**Description**
Decomposed Multi-Factor Model with tract-specific latent variables	Modification of the Decomposed Multi-Factor Model, in which the common variance of the same tracts in the two hemispheres is captured by tract-specific latent variables.
Decomposed Multi-Factor/Bifactor Model with tract-specific latent variables	Modification of the Decomposed Multi-Factor/Bifactor Model, in which the common variance of the same tracts in the two hemispheres is captured by tract-specific latent variables.
Decomposed Hierarchical Multi-Factor Model with tract-specific latent variables	Modification of the Decomposed Hierarchical Multi-Factor Model, in which the common variance of the same tracts in the two hemispheres is captured by tract-specific latent variables.
Decomposed Three-Level Multi-Factor Model with tract-specific latent variables	Modification of the Decomposed Three-Level Multi-Factor Model, in which the common variance of the same tracts in the two hemispheres is captured by tract-specific latent variables.
Decomposed Two-Level Multi-Factor Bifactor with tract-specific latent variables	Modification of the Decomposed Two-Level Multi-Factor Model, in which the common variance of the same tracts in the two hemispheres is captured by tract-specific latent variables.

Note: Each model is separately specified and estimated for the markers FA, INVF, and MTR. For identification of the models, we fixed the variances of all latent variables to 1 and freely estimated all factor loadings. If the variances of any manifest or latent variables were estimated to be negative without being statistically significant, we fixed the corresponding variance to 0.

Supplementary Table 2

Model fit indices FA for preregistered models (Dortmund data)

	chisq	df	pvalue	cfi	rmsea
Indicator-Only Model	10787.90	1326	0	0.00	0.22
Single-Factor Model	5790.95	1274	0	0.52	0.15
Bifactor Model	4438.50	1222	0	0.66	0.13
Hierarchical Model	5587.92	1273	0	0.54	0.15
Multi-Factor Model	NA	NA	NA	NA	NA
Multi-Factor/Bifactor Model	4218.63	1222	0	0.68	0.13
Hierarchical Multi-Factor Model	4894.94	1269	0	0.62	0.14
Three-Level Multi-Factor Model	5119.63	1269	0	0.59	0.14
Two-Level Multi-Factor Bifactor	NA	NA	NA	NA	NA
Decomposed Multi-Factor Model	4659.83	1253	0	0.64	0.14
Decomposed Multi-Factor/Bifactor Model	4105.65	1222	0	0.70	0.13
Decomposed Hierarchical Multi-Factor Model	NA	NA	NA	NA	NA
Decomposed Three-Level Multi-Factor Model	NA	NA	NA	NA	NA
Decomposed Two-Level Multi-Factor Bifactor	4062.20	1215	0	0.70	0.13
Indicator-Only Model with tract-specific latent variables	7027.71	1299	0	0.39	0.17
Single-Factor Model with tract-specific latent variables	4186.79	1273	0	0.69	0.12
Bifactor Model with tract-specific latent variables	3486.51	1247	0	0.76	0.11
Hierarchical Model with tract-specific latent variables	4116.41	1272	0	0.70	0.12
Multi-Factor Model with tract-specific latent variables	NA	NA	NA	NA	NA
Multi-Factor/Bifactor Model with tract-specific latent variables	3417.13	1248	0	0.77	0.11
Hierarchical Multi-Factor Model with tract-specific latent variables	4186.70	1268	0	0.69	0.12
Three-Level Multi-Factor Model with tract-specific latent variables	NA	NA	NA	NA	NA
Two-Level Multi-Factor Bifactor with tract-specific latent variables	3385.71	1244	0	0.77	0.11
Decomposed Multi-Factor Model with tract-specific latent variables	NA	NA	NA	NA	NA
Decomposed Multi-Factor/Bifactor Model with tract-specific latent variables	NA	NA	NA	NA	NA
Decomposed Hierarchical Multi-Factor Model with tract-specific latent variables	4186.71	1266	0	0.69	0.12
Decomposed Three-Level Multi-Factor Model with tract-specific latent variables	NA	NA	NA	NA	NA
Decomposed Two-Level Multi-Factor Bifactor with tract-specific latent variables	NA	NA	NA	NA	NA

Note: Models with NAs did not converge. Abbreviations: chisq = chi-squared test statistic, df = degrees of freedom, cfi = comparative fit index, rmsea = root mean square error of approximation.

Supplementary Table 3

Problems with preregistered models for the FA marker (Dortmund data)

Marker	Name of the model	Status	Lavaan Warning
FA	Multi-Factor Model	not converged	Model estimation FAILED! Returning starting values.
FA	Two-Level Multi-Factor Bifactor	not converged	Model estimation FAILED! Returning starting values.
FA	Decomposed Multi-Factor Model	converged	Covariance matrix of latent variables is not positive definite
FA	Decomposed Hierarchical Multi-Factor Model	not converged	Model estimation FAILED! Returning starting values.
FA	Decomposed Three-Level Multi-Factor Model	not converged	Model estimation FAILED! Returning starting values.
FA	Decomposed Two-Level Multi-Factor Bifactor	converged	The variance-covariance matrix of the estimated parameters (vcov) does not appear to be positive definite! The smallest eigenvalue (= -1.650975e+05) is smaller than zero. This may be a symptom that the model is not identified. Computation of robust CFI failed. Computation of robust RMSEA failed.
FA	Multi-Factor Model with tract-specific latent variables	not converged	Model estimation FAILED! Returning starting values.
FA	Three-Level Multi-Factor Model with tract-specific latent variables	not converged	Model estimation FAILED! Returning starting values.
FA	Decomposed Multi-Factor Model with tract-specific latent variables	not converged	Model estimation FAILED! Returning starting values.
FA	Decomposed Multi-Factor/Bifactor Model with tract-specific latent variables	not converged	Model estimation FAILED! Returning starting values.
FA	Decomposed Three-Level Multi-Factor Model with tract-specific latent variables	not converged	Model estimation FAILED! Returning starting values.
FA	Decomposed Two-Level Multi-Factor Bifactor with tract-specific latent variables	not converged	Model estimation FAILED! Returning starting values.

Note: Only models that produced warnings are listed in this table.

Supplementary Table 4

Model fit indices INVF for preregistered models (Dortmund data)

	chisq	df	pvalue	cfi	rmsea
Indicator-Only Model	20633.38	1326	0	0.00	0.31
Single-Factor Model	9976.08	1274	0	0.55	0.21
Bifactor Model	7371.88	1222	0	0.68	0.18
Hierarchical Model	8796.75	1273	0	0.61	0.12
Multi-Factor Model	NA	NA	NA	NA	NA
Multi-Factor/Bifactor Model	NA	NA	NA	NA	NA
Hierarchical Multi-Factor Model	NA	NA	NA	NA	NA
Three-level Multi-Factor Model	7832.57	1269	0	0.66	0.18
Two-level Multi-Factor Bifactor	NA	NA	NA	NA	NA
Decomposed Multi-Factor Model	NA	NA	NA	NA	NA
Decomposed Multi-Factor/Bifactor Model	NA	NA	NA	NA	NA
Decomposed Hierarchical Multi-Factor Model	NA	NA	NA	NA	NA
Decomposed Three-level Multi-Factor Model	NA	NA	NA	NA	NA
Decomposed Two-level Multi-Factor Bifactor	6512.83	1215	0	0.73	0.17
Indicator-Only model with tract-specific latent variables	13224.76	1299	0	0.38	0.25
Single-Factor model with tract-specific latent variables	NA	NA	NA	NA	NA
Bifactor Model with tract-specific latent variables	NA	NA	NA	NA	NA
Hierarchical Model with tract-specific latent variables	NA	NA	NA	NA	NA
Multi-Factor Model with tract-specific latent variables	NA	NA	NA	NA	NA
Multi-Factor/Bifactor Model with tract-specific latent variables	NA	NA	NA	NA	NA
Hierarchical Multi-Factor Model with tract-specific latent variables	NA	NA	NA	NA	NA
Three-level Multi-Factor Model with tract-specific latent variables	NA	NA	NA	NA	NA
Two-level Multi-Factor Bifactor with tract-specific latent variables	NA	NA	NA	NA	NA
Decomposed Multi-Factor Model with tract-specific latent variables	NA	NA	NA	NA	NA
Decomposed Multi-Factor/Bifactor Model with tract-specific latent variables	6365.63	1249	0	0.73	0.17
Decomposed Hierarchical Multi-Factor Model with tract-specific latent variables	NA	NA	NA	NA	NA
Decomposed Three-level Multi-Factor Model with tract-specific latent variables	NA	NA	NA	NA	NA
Decomposed Two-level Multi-Factor Bifactor with tract-specific latent variables	NA	NA	NA	NA	NA

Note: Models with NAs did not converge. Abbreviations: chisq = chi-squared test statistic, df = degrees of freedom, cfi = comparative fit index, rmsea = root mean square error of approximation.

Supplementary Table 5

Problems with preregistered models for the INVF marker (Dortmund data)

Marker	Name of the model	Status	Lavaan Warning
INVF	Indicator-Only Model	converged	The smallest eigenvalue of the EM estimated variance-covariance matrix (Sigma) is smaller than 1e-05. This may cause numerical instabilities. Interpret the results with caution.
INVF	Single-Factor Model	converged	The smallest eigenvalue of the EM estimated variance-covariance matrix (Sigma) is smaller than 1e-05.This may cause numerical instabilities. Interpret the results with caution.
INVF	Bifactor Model	converged	The smallest eigenvalue of the EM estimated variance-covariance matrix (Sigma) is smaller than 1e-05. This may cause numerical instabilities. Interpret the results with caution.
INVF	Hierarchical Model	converged	The smallest eigenvalue of the EM estimated variance-covariance matrix (Sigma) is smaller than 1e-05. This may cause numerical instabilities. Interpret the results with caution.
INVF	Multi-Factor Model	not converged	The smallest eigenvalue of the EM estimated variance-covariance matrix (Sigma) is smaller than 1e-05. This may cause numerical instabilities. Interpret the results with caution. Model estimation FAILED! Returning starting values..
INVF	Multi-Factor/Bifactor Model	not converged	The smallest eigenvalue of the EM estimated variance-covariance matrix (Sigma) is smaller than 1e-05. This may cause numerical instabilities. Interpret the results with caution. Model estimation FAILED! Returning starting values.
INVF	Hierarchical Multi-Factor Model	not converged	The smallest eigenvalue of the EM estimated variance-covariance matrix (Sigma) is smaller than 1e-05. This may cause numerical instabilities. Interpret the results with caution. Model estimation FAILED! Returning starting values.
INVF	Three-Level Multi-Factor Model	converged	The smallest eigenvalue of the EM estimated variance-covariance matrix (Sigma) is smaller than 1e-05. This may cause numerical instabilities. Interpret the results with caution.
INVF	Two-Level Multi-Factor Bifactor	not converged	The smallest eigenvalue of the EM estimated variance-covariance matrix (Sigma) is smaller than 1e-05. This may cause numerical instabilities. Interpret the results with caution. Model estimation FAILED! Returning starting values.
INVF	Decomposed Multi-Factor Model	not converged	The smallest eigenvalue of the EM estimated variance-covariance matrix (Sigma) is smaller than 1e-05. This may cause numerical instabilities. Interpret the results with caution. Model estimation FAILED! Returning starting values.
INVF	Decomposed Multi-Factor/Bifactor Model	not converged	The smallest eigenvalue of the EM estimated variance-covariance matrix (Sigma) is smaller than 1e-05. This may cause numerical instabilities. Interpret the results with caution. Model estimation FAILED! Returning starting values.

Problems with preregistered models for the INVF marker (Dortmund data) (continued)

Marker	Name of the model	Status	Lavaan Warning
INVF	Decomposed Hierarchical Multi-Factor Model	not converged	The smallest eigenvalue of the EM estimated variance-covariance matrix (Sigma) is smaller than 1e-05. This may cause numerical instabilities. Interpret the results with caution. Model estimation FAILED! Returning starting values.
INVF	Decomposed Three-Level Multi-Factor Model	not converged	The smallest eigenvalue of the EM estimated variance-covariance matrix (Sigma) is smaller than 1e-05. This may cause numerical instabilities. Interpret the results with caution. Model estimation FAILED! Returning starting values.
INVF	Decomposed Two-Level Multi-Factor Bifactor	converged	The smallest eigenvalue of the EM estimated variance-covariance matrix (Sigma) is smaller than 1e-05. This may cause numerical instabilities. Interpret the results with caution.The variance-covariance matrix of the estimated parameters (vcov) does not appear to be positive definite! The smallest eigenvalue (= -8.775824e+02) is smaller than zero. This may be a symptom that the model is not identified. Computation of robust CFI failed.Computation of robust RMSEA failed.
INVF	Indicator-Only Model with tract-specific latent variables	converged	The smallest eigenvalue of the EM estimated variance-covariance matrix (Sigma) is smaller than 1e-05. This may cause numerical instabilities. Interpret the results with caution.The variance-covariance matrix of the estimated parameters (vcov) does not appear to be positive definite! The smallest eigenvalue (= -8.775824e+02) is smaller than zero. This may be a symptom that the model is not identified.
INVF	Single-Factor Model with tract-specific latent variables	not converged	The smallest eigenvalue of the EM estimated variance-covariance matrix (Sigma) is smaller than 1e-05. This may cause numerical instabilities. Interpret the results with caution. Model estimation FAILED! Returning starting values.
INVF	Bifactor Model with tract-specific latent variables	not converged	The smallest eigenvalue of the EM estimated variance-covariance matrix (Sigma) is smaller than 1e-05. This may cause numerical instabilities. Interpret the results with caution. Model estimation FAILED! Returning starting values.
INVF	Hierarchical Model with tract-specific latent variables	not converged	The smallest eigenvalue of the EM estimated variance-covariance matrix (Sigma) is smaller than 1e-05. This may cause numerical instabilities. Interpret the results with caution. Model estimation FAILED! Returning starting values.
INVF	Multi-Factor Model with tract-specific latent variables	not converged	The smallest eigenvalue of the EM estimated variance-covariance matrix (Sigma) is smaller than 1e-05. This may cause numerical instabilities. Interpret the results with caution. Model estimation FAILED! Returning starting values.
INVF	Multi-Factor/Bifactor Model with tract-specific latent variables	not converged	The smallest eigenvalue of the EM estimated variance-covariance matrix (Sigma) is smaller than 1e-05. This may cause numerical instabilities. Interpret the results with caution. Model estimation FAILED! Returning starting values.

Problems with preregistered models for the INVF marker (Dortmund data) (continued)

Marker	Name of the model	Status	Lavaan Warning
INVF	Hierarchical Multi-Factor Model with tract-specific latent variables	not converged	The smallest eigenvalue of the EM estimated variance-covariance matrix (Sigma) is smaller than 1e-05. This may cause numerical instabilities. Interpret the results with caution. Model estimation FAILED! Returning starting values.
INVF	Three-Level Multi-Factor Model with tract-specific latent variables	not converged	The smallest eigenvalue of the EM estimated variance-covariance matrix (Sigma) is smaller than 1e-05. This may cause numerical instabilities. Interpret the results with caution. Model estimation FAILED! Returning starting values.
INVF	Two-Level Multi-Factor Bifactor with tract-specific latent variables	not converged	The smallest eigenvalue of the EM estimated variance-covariance matrix (Sigma) is smaller than 1e-05. This may cause numerical instabilities. Interpret the results with caution. Model estimation FAILED! Returning starting values.
INVF	Decomposed Multi-Factor Model with tract-specific latent variables	not converged	The smallest eigenvalue of the EM estimated variance-covariance matrix (Sigma) is smaller than 1e-05. This may cause numerical instabilities. Interpret the results with caution. Model estimation FAILED! Returning starting values.
INVF	Decomposed Multi-Factor/Bifactor Model with tract-specific latent variables	converged	The smallest eigenvalue of the EM estimated variance-covariance matrix (Sigma) is smaller than 1e-05. This may cause numerical instabilities. Interpret the results with caution.
INVF	Decomposed Hierarchical Multi-Factor Model with tract-specific latent variables	not converged	The smallest eigenvalue of the EM estimated variance-covariance matrix (Sigma) is smaller than 1e-05. This may cause numerical instabilities. Interpret the results with caution. Model estimation FAILED! Returning starting values.
INVF	Decomposed Three-Level Multi-Factor Model with tract-specific latent variables	not converged	The smallest eigenvalue of the EM estimated variance-covariance matrix (Sigma) is smaller than 1e-05. This may cause numerical instabilities. Interpret the results with caution. Model estimation FAILED! Returning starting values.
INVF	Decomposed Two-Level Multi-Factor Bifactor with tract-specific latent variables	not converged	The smallest eigenvalue of the EM estimated variance-covariance matrix (Sigma) is smaller than 1e-05. This may cause numerical instabilities. Interpret the results with caution. Model estimation FAILED! Returning starting values.

Note: Only models that produced warnings are listed in this table.

Supplementary Table 6

Model fit indices MTR for preregistered models (Dortmund data)

	chisq	df	pvalue	cfi	rmsea
Indicator-Bifactor Model	23167.89	1326	0	0.00	0.33
Single-Factor Model	8643.04	1274	0	0.66	0.20
Bifactor Model	7623.20	1222	0	0.71	0.19
Hierarchical Model	8616.56	1273	0	0.66	0.20
Multi-Factor Model	7937.80	1264	0	0.69	0.19
Multi-Factor/Bifactor Model	NA	NA	NA	NA	NA
Hierarchical Multi-Factor Model	8028.97	1269	0	0.69	0.19
Three-Level Multi-Factor Model	8028.97	1269	0	0.69	0.19
Two-Level Multi-Factor Bifactor	7236.11	1218	0	0.72	0.18
Decomposed Multi-Factor Model	7746.21	1253	0	0.70	0.19
Decomposed Multi-Factor/Bifactor Model	NA	NA	NA	NA	NA
Decomposed Hierarchical Multi-Factor Model	NA	NA	NA	NA	NA
Decomposed Three-Level Multi-Factor Model	NA	NA	NA	NA	NA
Decomposed Two-Level Multi-Factor Bifactor	NA	NA	NA	NA	NA
Indicator-Bifactor Model with tract-specific latent variables	16753.13	1299	0	0.29	0.28
Single-Factor Model with tract-specific latent variables	NA	NA	NA	NA	NA
Bifactor Model with tract-specific latent variables	NA	NA	NA	NA	NA
Hierarchical Model with tract-specific latent variables	NA	NA	NA	NA	NA
Multi-Factor Model with tract-specific latent variables	NA	NA	NA	NA	NA
Multi-Factor/Bifactor Model with tract-specific latent variables	7510.15	1248	0	0.71	0.18
Hierarchical Multi-Factor Model with tract-specific latent variables	NA	NA	NA	NA	NA
Three-Level Multi-Factor Model with tract-specific latent variables	NA	NA	NA	NA	NA
Two-Level Multi-Factor Bifactor with tract-specific latent variables	NA	NA	NA	NA	NA
Decomposed Multi-Factor Model with tract-specific latent variables	NA	NA	NA	NA	NA
Decomposed Multi-Factor/Bifactor Model with tract-specific latent variables	7624.83	1249	0	0.71	0.19
Decomposed Hierarchical Multi-Factor Model with tract-specific latent variables	NA	NA	NA	NA	NA
Decomposed Three-Level Multi-Factor Model with tract-specific latent variables	NA	NA	NA	NA	NA
Decomposed Two-Level Multi-Factor Bifactor with tract-specific latent variables	NA	NA	NA	NA	NA

Note: Models with NAs did not converge. Abbreviations: chisq = chi-squared test statistic, df = degrees of freedom, cfi = comparative fit index, rmsea = root mean square error of approximation.

Supplementary Table 7

Problems with preregistered models for the MTR marker (Dortmund data)

Marker	Name of the model	Status	Lavaan Warning
MTR	Multi-Factor/Bifactor Model	not converged	Model estimation FAILED! Returning starting values.
MTR	Decomposed Multi-Factor Model	converged	Covariance matrix of latent variables is not positive definite.
MTR	Multi-Factor/Bifactor Model	not converged	Model estimation FAILED! Returning starting values.
MTR	Hierarchical Multi-Factor Model	not converged	Model estimation FAILED! Returning starting values.
MTR	Three-Level Multi-Factor Model	not converged	Model estimation FAILED! Returning starting values.
MTR	Two-Level Multi-Factor Bifactor	not converged	Model estimation FAILED! Returning starting values.
MTR	Single-Factor Model with tract-specific latent variables	not converged	Model estimation FAILED! Returning starting values.
MTR	Bifactor Model with tract-specific latent variables	not converged	Model estimation FAILED! Returning starting values.
MTR	Hierarchical Model with tract-specific latent variables	not converged	Model estimation FAILED! Returning starting values.
MTR	Multi-Factor Model with tract-specific latent variables	not converged	Model estimation FAILED! Returning starting values.
MTR	Hierarchical Multi-Factor Model with tract-specific latent variables	not converged	Model estimation FAILED! Returning starting values.
MTR	Three-Level Multi-Factor Model with tract-specific latent variables	not converged	Model estimation FAILED! Returning starting values.
MTR	Two-Level Multi-Factor Bifactor with tract-specific latent variables	not converged	Model estimation FAILED! Returning starting values.
MTR	Multi-Factor Model with tract-specific latent variables	not converged	Model estimation FAILED! Returning starting values.
MTR	Hierarchical Multi-Factor Model with tract-specific latent variables	not converged	Model estimation FAILED! Returning starting values.
MTR	Three-Level Multi-Factor Model with tract-specific latent variables	not converged	Model estimation FAILED! Returning starting values.
MTR	Two-Level Multi-Factor Bifactor with tract-specific latent variables	not converged	Model estimation FAILED! Returning starting values.

Note: Only models that produced warnings are listed in this table.

Supplementary Figure 1

Measurement models 1

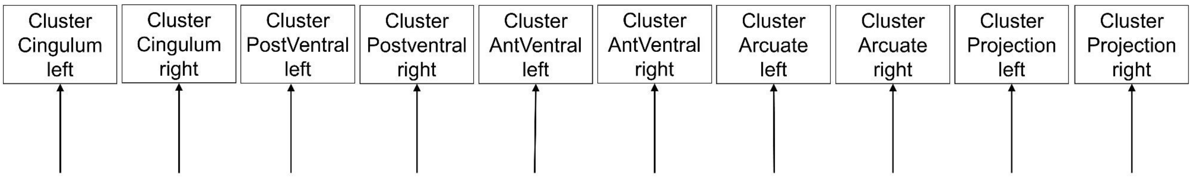

(a) *Clustered Indicator-Only Model (corresponds to the preregistered Indicator-Only Model)*

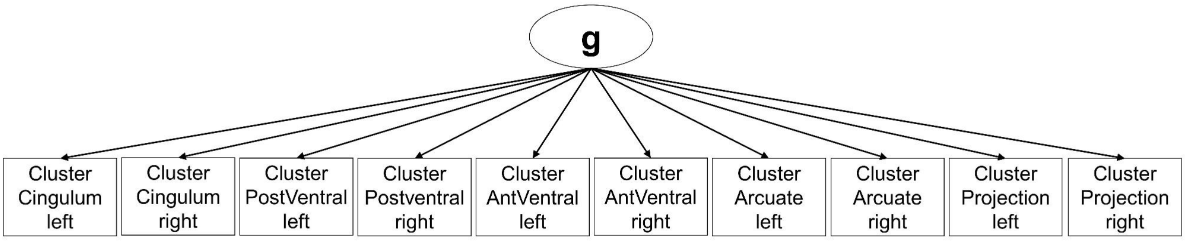

(b) *Clustered Single-Factor Model (corresponds to the preregistered Single-Factor Model)*

Note. The average marker values of ten functional clusters of 52 tracts are included as independent manifest variables and no general factor is modeled (a). A general factor loads onto the ten functional cluster-average indicators (b). Residual errors and the data source regressor are not depicted for the Clustered Single-Factor Model for simplification.

Supplementary Figure 2

Measurement models 2

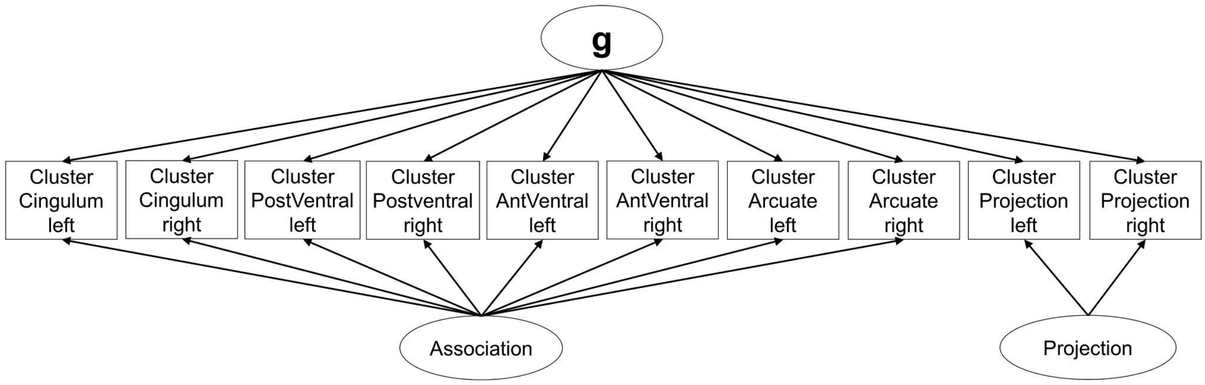

(a) *Clustered Bifactor Model (corresponds to the preregistered Bifactor Model)*

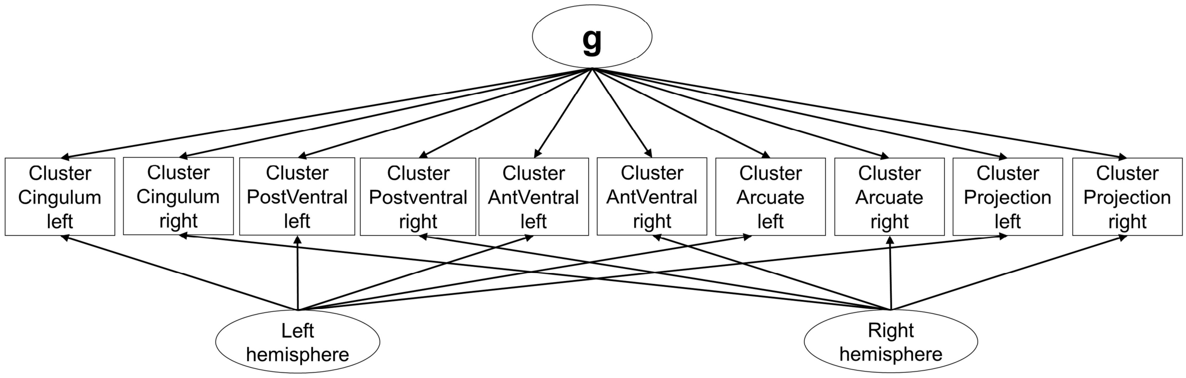

(b) *Clustered Hemisphere-Bifactor Model (exploratory model)*

Note. A general factor is included as a primary dimension and two latent variables (association and projection fibers) are included as a secondary dimension (a). A general factor is included as a primary dimension and two latent variables (left and right hemisphere) are included as a secondary dimension (b). Residual errors and the data source regressor are not depicted for simplification.

Supplementary Figure 3

Measurement models 3

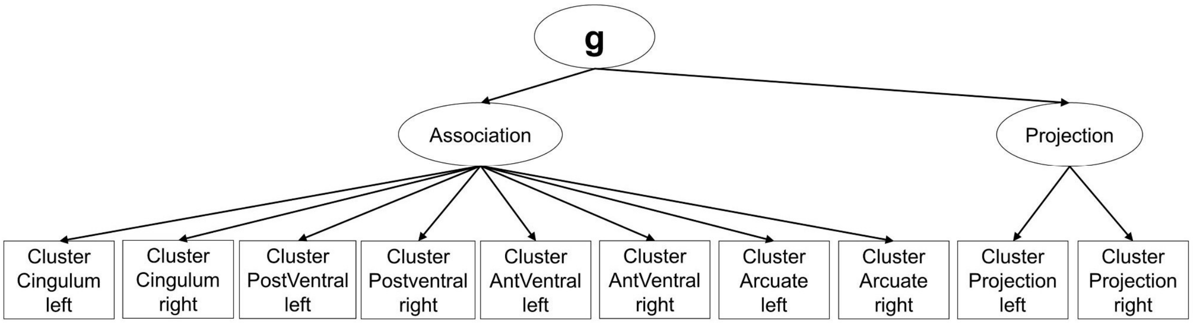

(a) *Clustered Hierarchical Model (corresponds to the preregistered Hierarchical Model)*

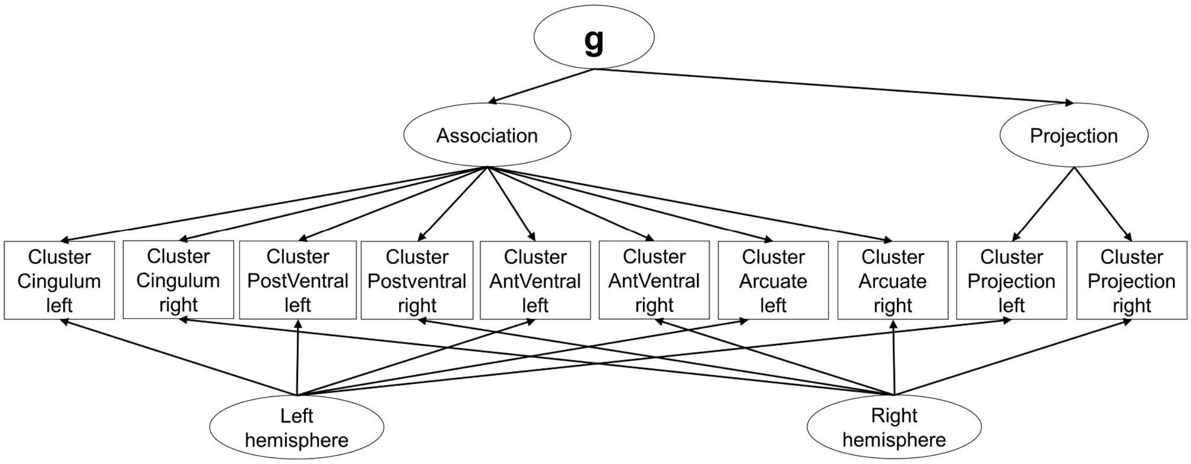

(b) *Clustered Hierarchical Hemisphere-Bifactor Model (exploratory model)*

Note. The general factor loads onto a latent variable association fibers and a latent variable projection fibers, which in turn load onto the ten functional cluster-average indicators (a). Similar to the Clustered Hemisphere-Bifactor Model but two latent factors for association and projection fibers are included as an additional hierarchy level (b). Residual errors and the data source regressor are not depicted for simplification.

Supplementary Figure 4

Measurement models 4

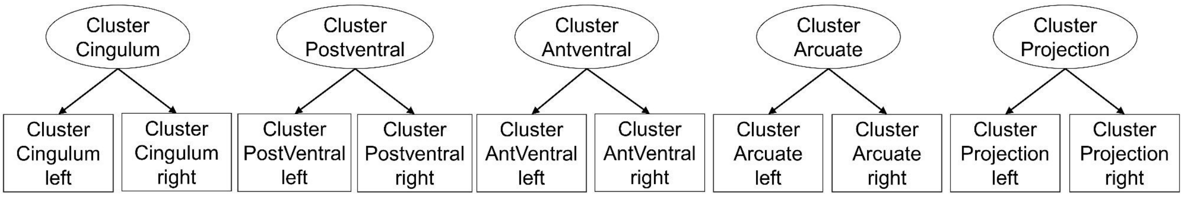

(a) *Bilateral Clustered Indicator-Only Model (corresponds to the preregistered Indicator-Only Model with tract-specific latent variables)*

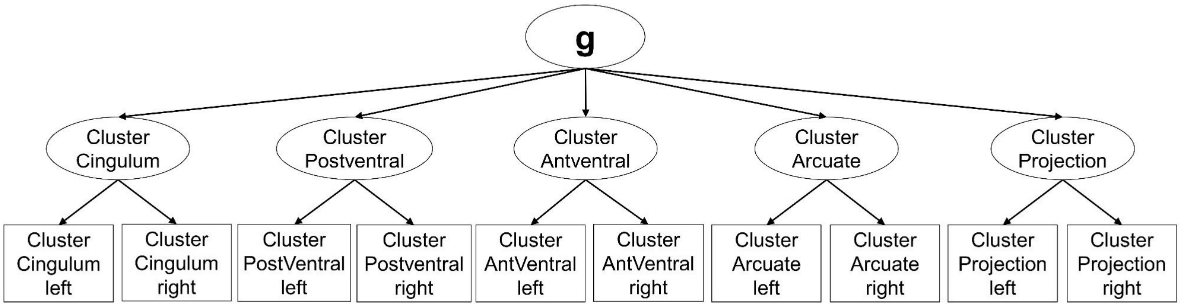

(b) *Bilateral Clustered Single-Factor Model (corresponds to the preregistered Single-Factor Model with tract-specific latent variables)*

Note. Modification of the Clustered Indicator-Only Model with bilateral latent cluster variables (a). Modification of the Clustered Single-Factor Model with bilateral latent cluster variables (b). Residual errors and the data source regressor are not depicted for the Clustered Single-Factor Model for simplification.

Supplementary Figure 5

Measurement models 5

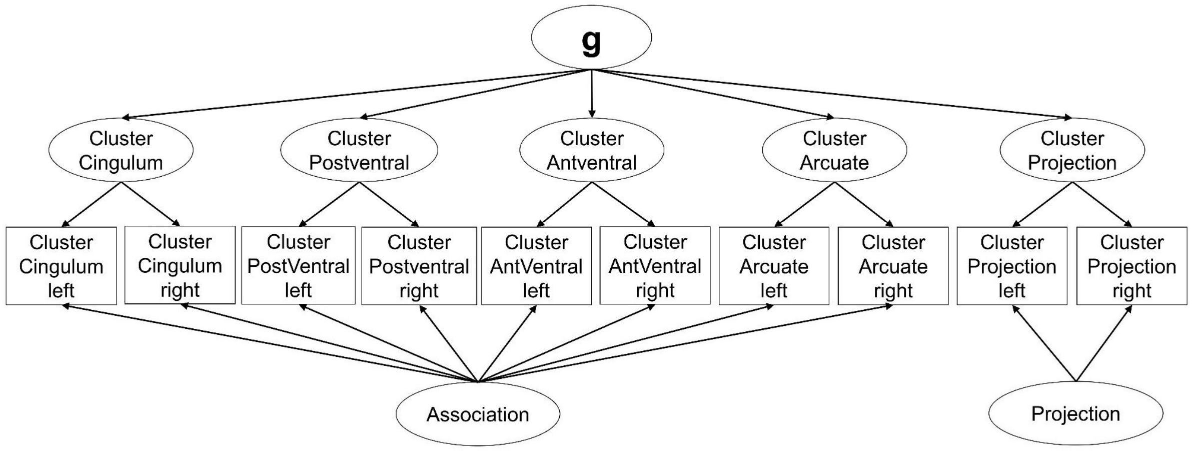

(a) *Bilateral Clustered Bifactor Model (corresponds to the preregistered Bifactor Model with tract-specific latent variables)*

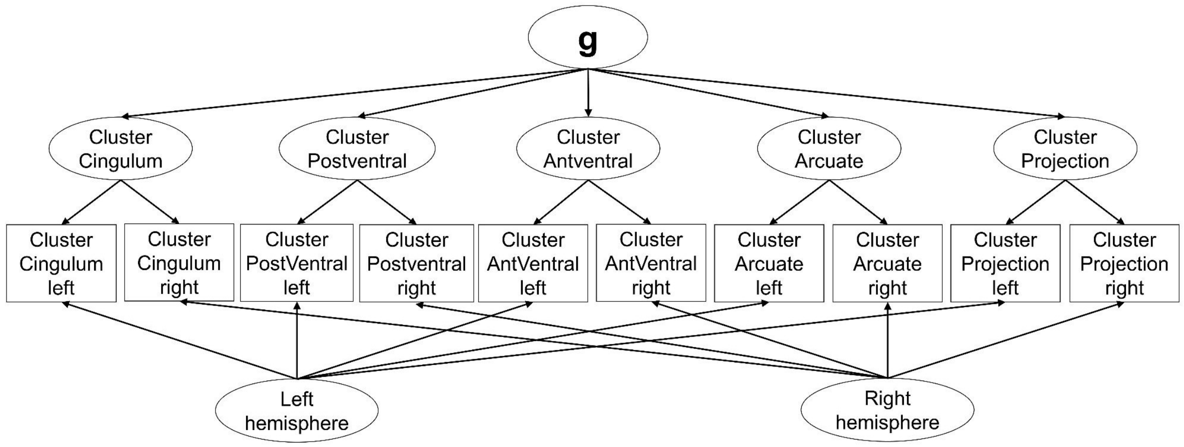

(b) *Bilateral Clustered Hemisphere-Bifactor Model (exploratory model)*

Note. Modification of the Clustered Bifactor Model with bilateral latent cluster variables (a). Modification of the Clustered Hemisphere-Bifactor Model with bilateral latent cluster variables (b). Residual errors and the data source regressor are not depicted for simplification.

Supplementary Figure 6

Measurement models 6

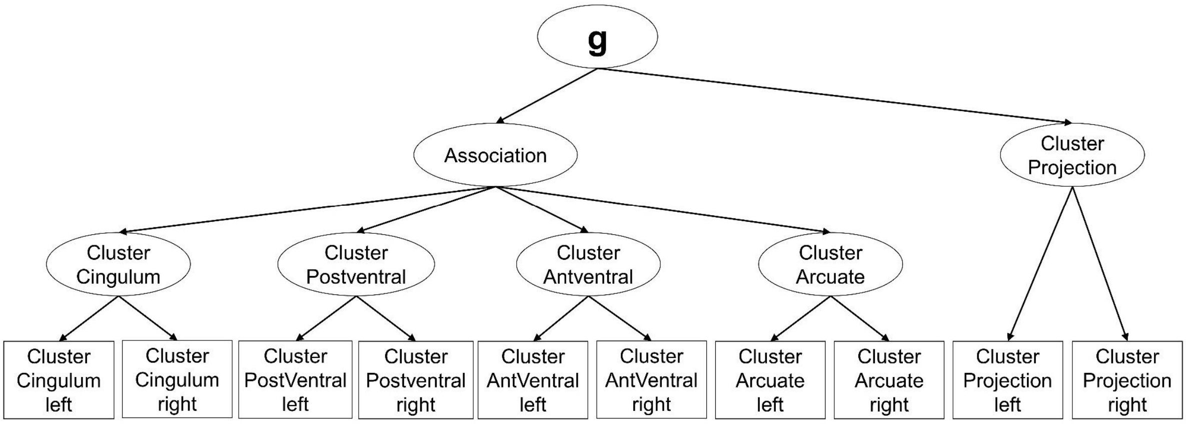

(a) *Bilateral Clustered Hierarchical Model (corresponds to the preregistered Hierarchical Model with tract-specific latent variables)*

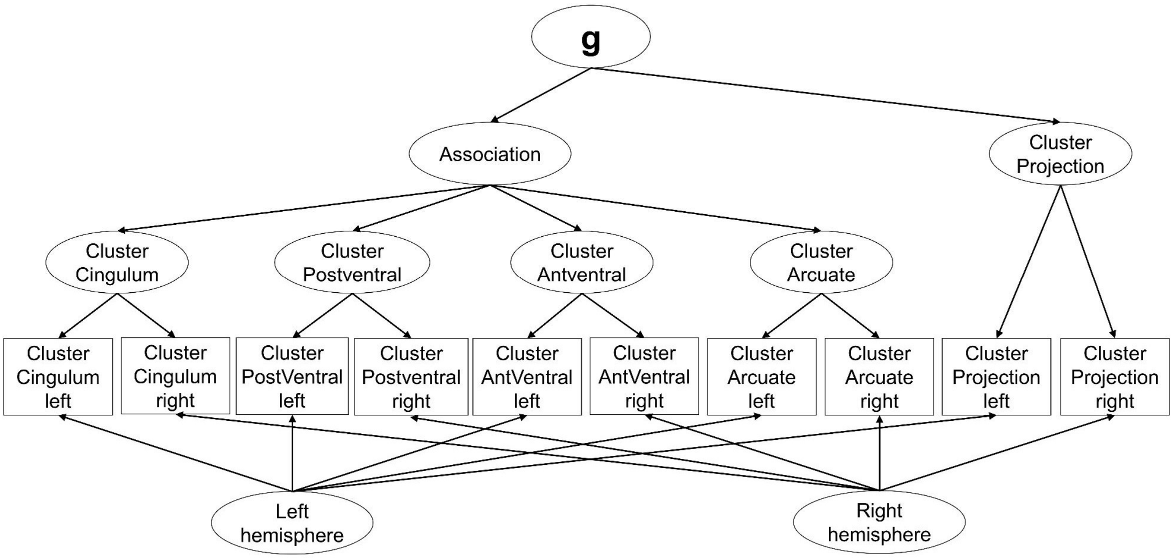

(b) *Bilateral Clustered Hierarchical Hemisphere-Bifactor Model (exploratory model)*

Note. Modification of the Clustered Hierarchical Model with bilateral latent cluster variables (a). Modification of the Clustered Hierarchical Hemisphere-Bifactor Model with bilateral latent cluster variables (b). Residual errors and the data source regressor are not depicted for simplification.

Supplementary Table 8

Model fit indices FA

	chisq	df	pvalue	cfi	rmsea	tli	srmr	aic
Clustered Indicator-Only Model	3587.79	45	0	0.00	0.46	-0.23	0.51	9922.96
Clustered Single-Factor Model	745.58	44	0	0.80	0.21	0.75	0.05	7082.75
Clustered Bifactor Model	288.20	33	0	0.93	0.15	0.88	0.03	6647.38
Clustered Hemisphere-Bifactor Model	687.03	34	0	0.82	0.23	0.70	0.06	7044.21
Clustered Hierarchical Model	546.77	42	0	0.86	0.18	0.81	0.05	6887.94
Clustered Hierarchical Hemisphere-Bifactor Model	438.54	32	0	0.88	0.18	0.80	0.05	6799.72
Bilateral Clustered Indicator-Only Model	1428.27	45	0	0.61	0.29	0.52	0.46	7763.45
Bilateral Clustered Single-Factor Model	160.85	39	0	0.97	0.09	0.95	0.08	6508.02
Bilateral Clustered Bifactor Model	NA	NA	NA	NA	NA	NA	NA	NA
**Bilateral Clustered Hemisphere-Bifactor Model**	**103.91**	**28**	**0**	**0.98**	**0.09**	**0.96**	**0.07**	**6473.08**
Bilateral Clustered Hierarchical Model	160.85	38	0	0.97	0.09	0.95	0.08	6510.02
Bilateral Clustered Hierarchical Hemisphere-Bifactor Model	103.91	27	0	0.98	0.09	0.96	0.07	6475.08

Note: Models with NAs did not converge. The winning measurement models are highlighted in bold letters.

Abbreviations: chisq = chi-squared test statistic, df = degrees of freedom, cfi = Comparative Fit Index, rmsea = Root Mean Square Error of Approximation, tli = Tucker-Lewis Index, srmr = Standardized Root Mean Square Residual, aic = Akaike Information Criterion.

Supplementary Table 9

Model fit indices INVF

	chisq	df	pvalue	cfi	rmsea	tli	srmr	aic
Clustered Indicator-Only Model	7099.04	45	0	0.00	0.66	-0.22	0.66	9908.77
Clustered Single-Factor Model	1588.87	44	0	0.78	0.31	0.73	0.03	4400.61
Clustered Bifactor Model	732.92	33	0	0.90	0.24	0.83	0.02	3566.65
Clustered Hemisphere-Bifactor Model	1293.71	32	0	0.82	0.33	0.69	0.05	4129.45
Clustered Hierarchical Model	1264.10	42	0	0.83	0.28	0.78	0.03	4079.83
Clustered Hierarchical Hemisphere-Bifactor Model	862.10	32	0	0.88	0.27	0.80	0.06	3697.83
Bilateral Clustered Indicator-Only Model	3118.75	45	0	0.56	0.43	0.47	0.62	5928.48
Bilateral Clustered Single-Factor Model	683.20	39	0	0.91	0.21	0.87	0.02	3504.93
Bilateral Clustered Bifactor Model	551.40	34	0	0.93	0.20	0.88	0.02	3383.13
**Bilateral Clustered Hemisphere-Bifactor Model**	**162.71**	**29**	**0**	**0.98**	**0.11**	**0.96**	**0.03**	**3004.44**
Bilateral Clustered Hierarchical Model	683.20	38	0	0.91	0.22	0.87	0.02	3506.93
Bilateral Clustered Hierarchical Hemisphere-Bifactor Model	172.85	28	0	0.98	0.11	0.96	0.04	3016.58

Note: Models with NAs did not converge. The winning measurement model is highlighted in bold letters.

Abbreviations: chisq = chi-squared test statistic, df = degrees of freedom, cfi = Comparative Fit Index, rmsea = Root Mean Square Error of Approximation, tli = Tucker-Lewis Index, srmr = Standardized Root Mean Square Residual, aic = Akaike Information Criterion.

Supplementary Table 10

Model fit indices MTR (Dortmund Data)

	chisq	df	pvalue	cfi	rmsea	tli	srmr	aic
Clustered Indicator-Only Model	3514.29	45	0	0	0.72	0	0.77	1741.48
Clustered Single-Factor Model	308.37	35	0	0.92	0.23	0.90	0.01	-1444.44
Clustered Bifactor Model	264.9132	26	0	0.93	0.25	0.88	0.01	-1469.90
Clustered Hemisphere-Bifactor Model	187.77	27	0	0.95	0.20	0.92	0.01	-1549.04
Clustered Hierarchical Model	NA	NA	NA	NA	NA	NA	NA	NA
Clustered Hierarchical Hemisphere-Bifactor Model	175.48	27	0	0.96	0.19	0.93	0.01	-1561.33
Bilateral Clustered Indicator-Only Model	1951.42	40	0	0.45	0.57	0.38	0.73	188.61
Bilateral Clustered Single-Factor Model	NA	NA	NA	NA	NA	NA	NA	NA
Bilateral Clustered Bifactor Model	NA	NA	NA	NA	NA	NA	NA	NA
**Bilateral Clustered Hemisphere-Bifactor Model**	**108.61**	**27**	**0**	**0.98**	**0.14**	**0.96**	**0.03**	**-1628.20**
Bilateral Clustered Hierarchical Model	NA	NA	NA	NA	NA	NA	NA	NA
**Bilateral Clustered Hierarchical Hemisphere-Bifactor Model**	**108.61**	**27**	**0**	**0.98**	**0.14**	**0.96**	**0.03**	**-1628.20**

Note: Models with NAs did not converge. The winning measurement model is highlighted in bold letters.

Abbreviations: chisq = chi-squared test statistic, df = degrees of freedom, cfi = Comparative Fit Index, rmsea = Root Mean Square Error of Approximation, tli = Tucker-Lewis Index, srmr = Standardized Root Mean Square Residual, aic = Akaike Information Criterion.

Supplementary Table 11

Model fit indices of the comprehensive white matter microstructure models

	chisq	df	pvalue	cfi	rmsea	tli	srmr	aic
**Combined Model (BiClusHem-BiF Model for all markers)**	**1457.90**	**391**	**0**	**0.93**	**0.09**	**0.91**	**0.08**	**10303.95**
Combined Model with g factor (BiClusHem-BiF for all markers)	1457.90	387	0	0.93	0.09	0.91	0.08	10311.95
Combined Model (HiBiClusHem-BiF Model for MTR)	1458.44	391	0	0.93	0.09	0.91	0.08	10304.49
Combined Model with g factor (HiBiClusHem-BiF Model for MTR)	1458.44	387	0	0.93	0.09	0.91	0.08	10312.49

Note: Models with NAs did not converge. The winning measurement model is highlighted in bold letters.

Abbreviations: chisq = chi-squared test statistic, df = degrees of freedom, cfi = Comparative Fit Index, rmsea = Root Mean Square Error of Approximation, tli = Tucker-Lewis Index, srmr = Standardized Root Mean Square Residual, aic = Akaike Information Criterion.

Supplementary Table 12

Model fit indices FA controlled for age

	chisq	df	pvalue	cfi	rmsea	tli	srmr	aic
Clustered Indicator-Only Model	3376.67	45	0	0.07	0.45	-0.34	0.42	9654.61
Clustered Single-Factor Model	785.00	53	0	0.80	0.19	0.75	0.06	7046.94
Clustered Bifactor Model	416.09	40	0	0.90	0.16	0.83	0.04	6704.94
Clustered Hemisphere-Bifactor Model	718.54	39	0	0.81	0.22	0.69	0.05	7008.48
Clustered Hierarchical Model	585.72	49	0	0.85	0.17	0.80	0.05	6855.65
Clustered Hierarchical Hemisphere-Bifactor Model	474.07	37	0	0.88	0.18	0.79	0.05	6768.01
Bilateral Clustered Indicator-Only Model	1347.94	50	0	0.64	0.27	0.53	0.38	7615.88
Bilateral Clustered Single-Factor Model	165.38	43	0	0.97	0.09	0.95	0.07	6447.31
Bilateral Clustered Bifactor Model	142.53	37	0	0.97	0.09	0.95	0.07	6436.47
**Bilateral Clustered Hemisphere-Bifactor Model**	**104.47**	**30**	**0**	**0.98**	**0.08**	**0.96**	**0.06**	**6412.40**
Bilateral Clustered Hierarchical Model	165.38	41	0	0.97	0.09	0.95	0.07	6451.31
Bilateral Clustered Hierarchical Hemisphere-Bifactor Model	100.67	27	0	0.98	0.09	0.95	0.05	6414.61

Note: Models with NAs did not converge. The winning measurement model is highlighted in bold letters.

Abbreviations: chisq = chi-squared test statistic, df = degrees of freedom, cfi = Comparative Fit Index, rmsea = Root Mean Square Error of Approximation, tli = Tucker-Lewis Index, srmr = Standardized Root Mean Square Residual, aic = Akaike Information Criterion.

Supplementary Table 13

Model fit indices INVF controlled for age

	chisq	df	pvalue	cfi	rmsea	tli	srmr	aic
Clustered Indicator-Only Model	7065.58	45	0	0.01	0.65	-0.42	0.60	9835.82
Clustered Single-Factor Model	1637.21	53	0	0.78	0.29	0.73	0.04	4391.45
Clustered Bifactor Model	751.26	40	0	0.90	0.22	0.84	0.02	3531.50
Clustered Hemisphere-Bifactor Model	1290.89	39	0	0.82	0.30	0.71	0.04	4073.13
Clustered Hierarchical Model	1285.48	49	0	0.83	0.26	0.77	0.03	4047.72
Clustered Hierarchical Hemisphere-Bifactor Model	870.11	37	0	0.88	0.25	0.79	0.05	3656.35
Bilateral Clustered Indicator-Only Model	3131.02	50	0	0.57	0.41	0.44	0.56	5891.27
Bilateral Clustered Single-Factor Model	675.30	43	0	0.91	0.20	0.87	0.02	3449.54
Bilateral Clustered Bifactor Model	557.45	37	0	0.93	0.20	0.87	0.01	3343.68
**Bilateral Clustered Hemisphere-Bifactor Model**	**149.78**	**30**	**0**	**0.98**	**0.10**	**0.96**	**0.02**	**2950.02**
Bilateral Clustered Hierarchical Model	675.30	41	0	0.91	0.21	0.86	0.02	3453.54
Bilateral Clustered Hierarchical Hemisphere-Bifactor Model	149.78	28	0	0.98	0.11	0.96	0.02	2954.02

Note: Models with NAs did not converge. The winning measurement model is highlighted in bold letters.

Abbreviations: chisq = chi-squared test statistic, df = degrees of freedom, cfi = Comparative Fit Index, rmsea = Root Mean Square Error of Approximation, tli = Tucker-Lewis Index, srmr = Standardized Root Mean Square Residual, aic = Akaike Information Criterion.

Supplementary Table 14

Model fit indices MTR controlled for age

	chisq	df	pvalue	cfi	rmsea	tli	srmr	aic
Clustered Indicator-Only Model	3523.55	45	0	0.00	0.72	-0.22	0.70	1748.96
Clustered Single-Factor Model	328.56	44	0	0.92	0.21	0.90	0.01	-1443.73
Clustered Bifactor Model	NA	NA	NA	NA	NA	NA	NA	NA
Clustered Hemisphere-Bifactor Model	205.21	34	0	0.95	0.18	0.92	0.01	-1547.38
Clustered Hierarchical Model	NA	NA	NA	NA	NA	NA	NA	NA
Clustered Hierarchical Hemisphere-Bifactor Model	193.11	34	0	0.95	0.18	0.93	0.01	-1559.47
Bilateral Clustered Indicator-Only Model	1966.90	45	0	0.45	0.53	0.33	0.66	192.32
Bilateral Clustered Single-Factor Model	NA	NA	NA	NA	NA	NA	NA	NA
Bilateral Clustered Bifactor Model	NA	NA	NA	NA	NA	NA	NA	NA
**Bilateral Clustered Hemisphere-Bifactor Model**	**127.75**	**34**	**0**	**0.97**	**0.14**	**0.96**	**0.03**	**-1624.83**
Bilateral Clustered Hierarchical Model	NA	NA	NA	NA	NA	NA	NA	NA
**Bilateral Clustered Hierarchical Hemisphere-Bifactor Model**	**127.71**	**34**	**0**	**0.97**	**0.14**	**0.96**	**0.03**	**-1624.87**

Note: Models with NAs did not converge. The winning measurement model is highlighted in bold letters.

Abbreviations: chisq = chi-squared test statistic, df = degrees of freedom, cfi = Comparative Fit Index, rmsea = Root Mean Square Error of Approximation, tli = Tucker-Lewis Index, srmr = Standardized Root Mean Square Residual, aic = Akaike Information Criterion.

Supplementary Figure 7

Pairwise correlations of the cluster-wise FA and MTR values in the Dortmund data

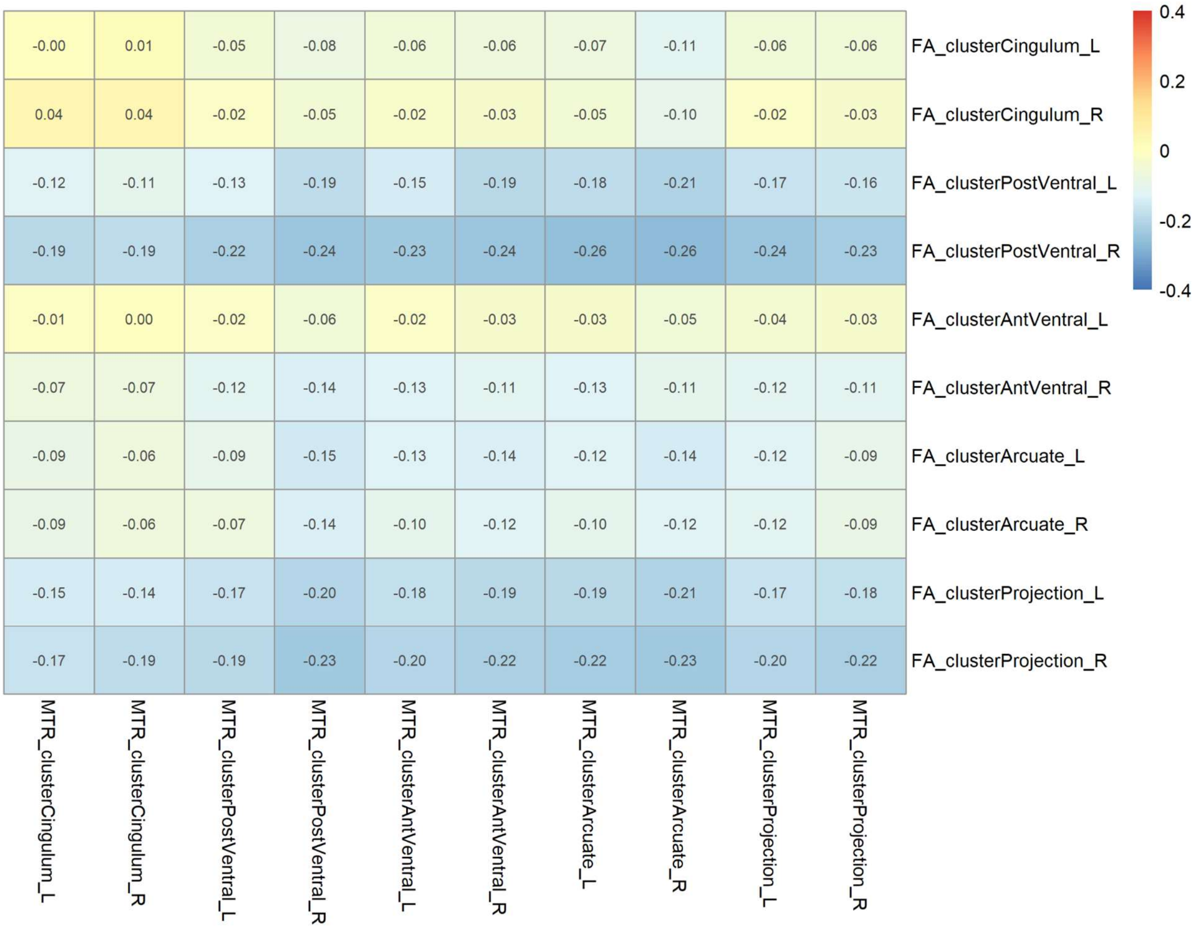

Note. Excerpt of the correlation matrix of all cluster-average FA and MTR calculated for the Dortmund data depicting the pairwise correlations between cluster-wise FA and MTR.

Abbreviations: AntVentral = anterior ventral system, PostVentral = posterior ventral system, FA = fractional anisotropy, MTR = magnetization transfer ratio, R = right hemisphere, L = left hemisphere.

Supplementary Figure 8

Pairwise partial correlations of the cluster-wise FA and INVF values controlled for data source

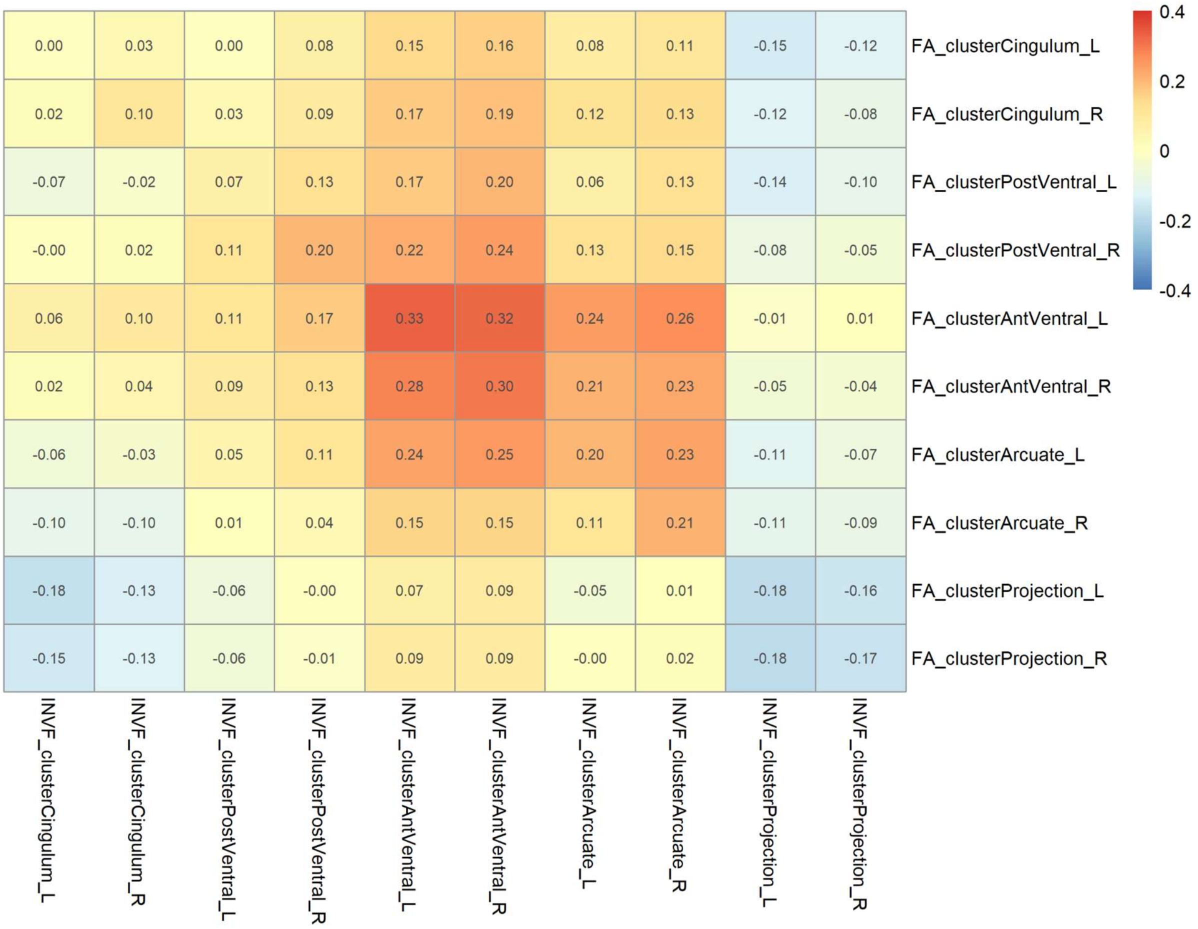

Note. Excerpt of the partial correlation matrix of all cluster-average FA and INVF values calculated for the combined data controlled for data source depicting the pairwise partial correlations between cluster-wise FA and INVF.

Abbreviations: AntVentral = anterior ventral system, PostVentral = posterior ventral system, FA = fractional anisotropy, INVF = intraneurite volume fraction, R = right hemisphere, L = left hemisphere.

Supplementary Figure 9

Pairwise correlations of the tract-wise FA and MTR values in the Dortmund data

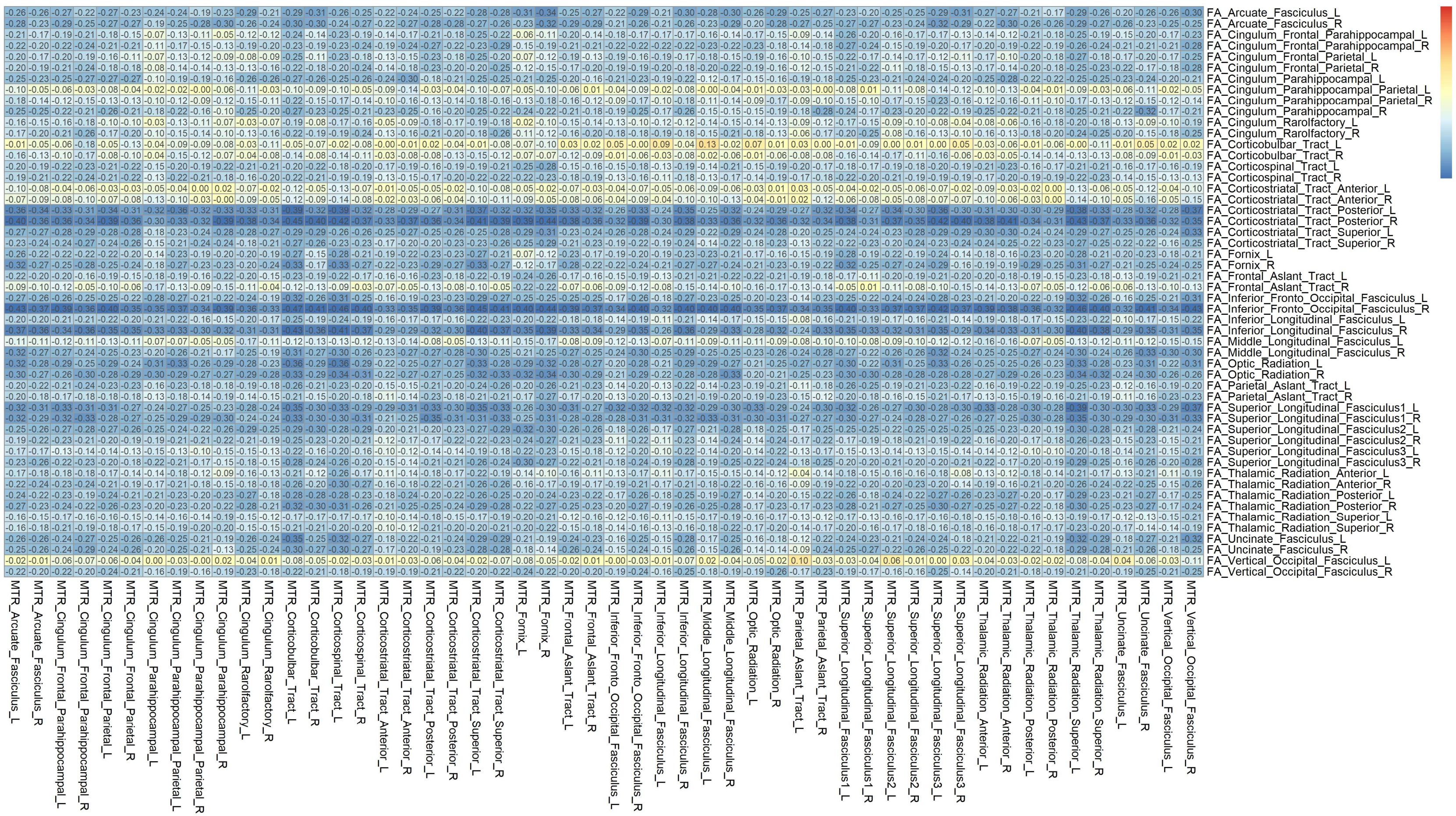

Note. Excerpt of the correlation matrix of all tract-average FA and MTR values calculated for the Dortmund data depicting the pairwise correlations between tract-wise FA and INVF.

Abbreviations: FA = fractional anisotropy, MTR = magnetization transfer ratio, R = right hemisphere, L = left hemisphere.

Supplementary Figure 10

Pairwise partial correlations of the tract-wise FA and INVF values controlled for data source

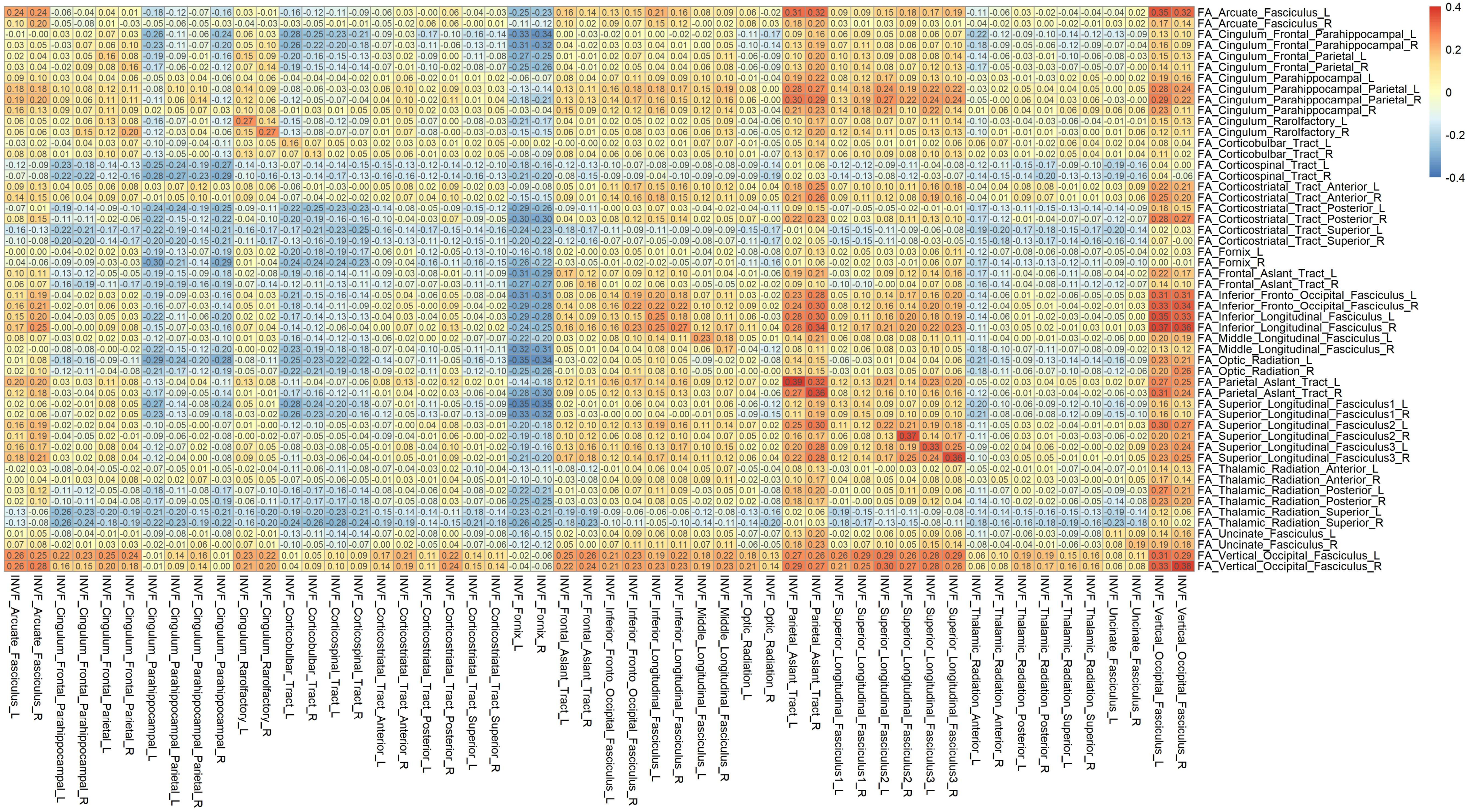

Note. Excerpt of the partial correlation matrix of all tract-average FA and INVF values calculated for the combined data controlled for data source depicting the pairwise correlations between tract-wise FA and INVF.

Abbreviations: FA = fractional anisotropy, INVF = intraneurite volume fraction, R = right hemisphere, L = left hemisphere.

Supplementary Figure 11

Pairwise correlations between FA and MTR in the ten tracts with lowest average ODI vs. in the ten tracts with highest average ODI

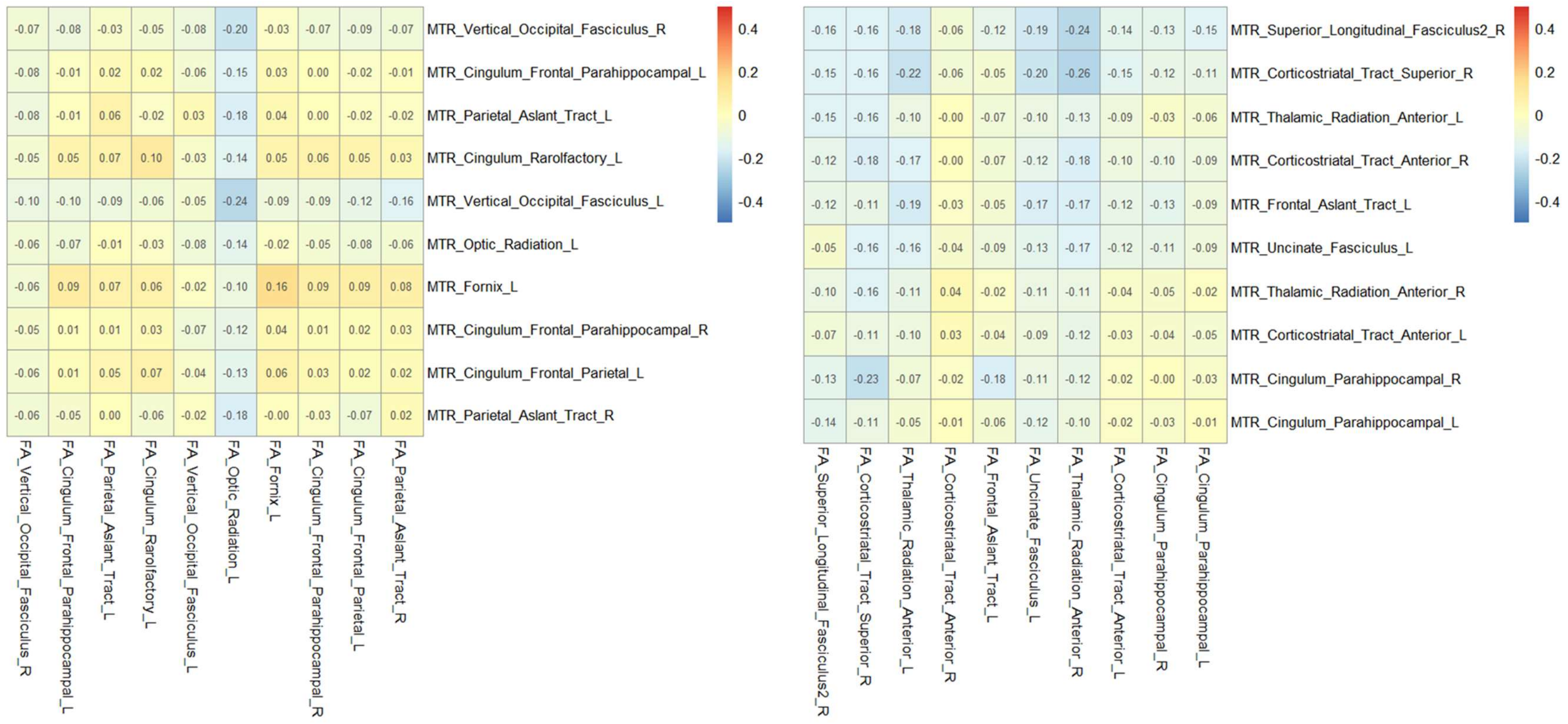

Note. Excerpt of the correlation matrix of all tract-average FA and INVF values calculated for the Dortmund data depicting the pairwise correlations between tract-wise FA and MTR in the ten lowest average ODI tracts (left) and in the ten highest average ODI tracts (right).

Abbreviations: FA = fractional anisotropy, INVF = intraneurite volume fraction, ODI = orientation dispersion index, R = right hemisphere, L = left hemisphere.

Supplementary Figure 12

Pairwise partial correlations between FA and INVF in the ten tracts with lowest average ODI vs. in the ten tracts with highest average ODI

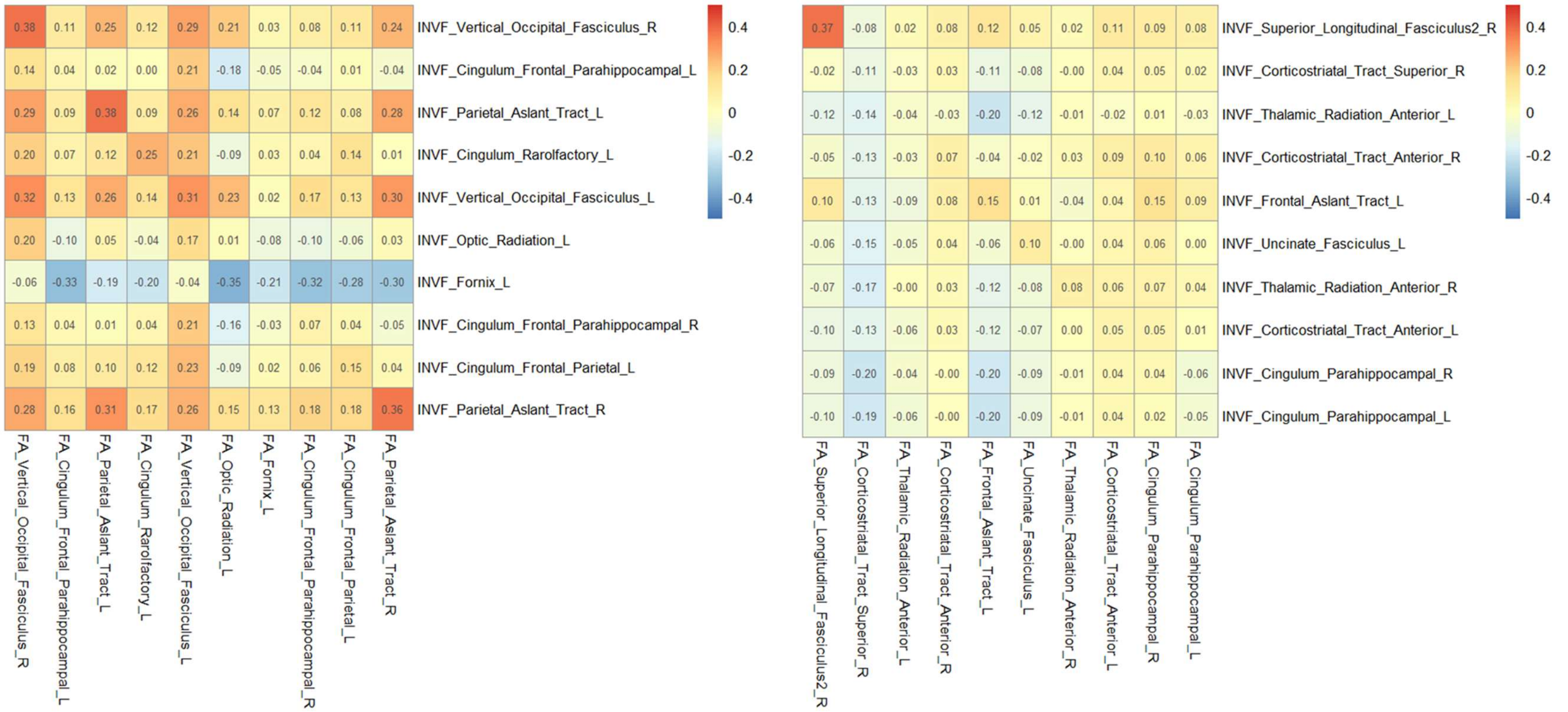

Note. Excerpt of the partial correlation matrix of all tract-average FA and INVF values calculated for the combined data controlled for data source depicting the pairwise partial correlations between tract-wise FA and INVF in the ten lowest average ODI tracts (left) and in the ten highest average ODI tracts (right).

Abbreviations: FA = fractional anisotropy, INVF = intraneurite volume fraction, ODI = orientation dispersion index, R = right hemisphere, L = left hemisphere.
